# Conceptual Modeling of mRNA Decay Provokes New Hypotheses

**DOI:** 10.1371/journal.pone.0107085

**Published:** 2014-09-25

**Authors:** Judith Somekh, Gal Haimovich, Adi Guterman, Dov Dori, Mordechai Choder

**Affiliations:** 1 Faculty of Industrial Engineering and Management, Technion - Israel Institute of Technology, Haifa, Israel; 2 Faculty of Medicine, Technion - Israel Institute of Technology, Haifa, Israel; 3 Engineering Systems Division, Massachusetts Institute of Technology, Cambridge, Massachusetts, United States of America; Korea University, Republic of Korea

## Abstract

Biologists are required to integrate large amounts of data to construct a working model of the system under investigation. This model is often informal and stored mentally or textually, making it prone to contain undetected inconsistencies, inaccuracies, or even contradictions, not much less than a representation in free natural language. Using Object-Process Methodology (OPM), a formal yet visual and humanly accessible conceptual modeling language, we have created an executable working model of the mRNA decay process in *Saccharomyces cerevisiae*, as well as the import of its components to the nucleus following mRNA decay. We show how our model, which incorporates knowledge from 43 articles, can reproduce outcomes that match the experimental findings, evaluate hypotheses, and predict new possible outcomes. Moreover, we were able to analyze the effects of the mRNA decay model perturbations related to gene and interaction deletions, and predict the nuclear import of certain decay factors, which we then verified experimentally. In particular, we verified experimentally the hypothesis that Rpb4p, Lsm1p, and Pan2p remain bound to the RNA 3′-untralslated region during the entire process of the 5′ to 3′ degradation of the RNA open reading frame. The model has also highlighted erroneous hypotheses that indeed were not in line with the experimental outcomes. Beyond the scientific value of these specific findings, this work demonstrates the value of the conceptual model as an *in silico* vehicle for hypotheses generation and testing, which can reinforce, and often even replace, risky, costlier wet lab experiments.

## Introduction

Formidable amounts of detailed pieces of knowledge regarding the structure and function of the living cell have been accumulating at an ever-growing rate. Biologists are required to integrate this large quantity of data to construct a working model of the system under investigation. Biological working models are conceived, stored, and managed mentally by biological researchers, and then expressed textually in natural language as the backbone of the published experimental results. Based on natural language, these mental working models are prone to inconsistencies and lack of completeness in their description of underlying biological mechanisms. Many questions that biologists may have can be better formulated and answered if data and information are converted into meaningful knowledge that is organized in an accessible, navigable formal model. Such organized and managed model-based knowledge is a critical stepping stone to gaining consistent understanding of how biological system–organisms–perform their top-level function of sustaining life. Moreover, a consistent model can potentially save expensive resources and precious time in performing unnecessary, duplicative, or inefficient experiments.

Using the currently available and the ever-growing quantities of information to create knowledge that will help understand normal and pathological biological processes and apply them in medicine mandates that information from seemingly disparate domains be assembled systematically to create a coherent system view. A conceptual, executable model, qualitatively describing the mechanisms underlying the operation of the biological system at various levels of detail would facilitate system-level comprehension by providing a consistent view of the system under study and enabling new hypotheses generation. These hypotheses can then be evaluated, verified or refuted by wet lab experiments.

Wet lab experiments, which often require the use of hazardous and costly materials and animals, can often linger for many months and may need to be repeated for various reasons, until a consistent result that is either supporting or disproving a hypothesis, is achieved. It is therefore of utmost importance to direct these high-risk experimental endeavors to the most promising avenues of investigation. Such avenues can be guided by a detailed formal conceptual model, in which all the knowledge about the system and its mechanisms is represented with high fidelity. As we show in this work, such a model can be valuable for generating and testing research hypotheses that can direct the experimental effort to promising directions, avoiding duplicative experiments or those that the model predicts would fail.

The areas of executable biology [1] have evolved to enable execution of complex biological systems using computational tools. These approaches enable simulating the dynamics of biological systems without the need to incorporate mathematical equations or details regarding compound quantities, which are either missing or masking the qualitative nature of the model. Indeed, formal executable models have been shown [1, 2] to be valuable in pinpointing where research should focus based on their ability to generate predictions and analyze the temporal aspects of the biological system. While several executable approaches are available, rather than providing an integrated view of the overall function, structure, and behavior of the system being modeled, many of them cover only partial aspects of the knowledge, such as gene expression relations, molecular interactions, processes or event-related states.

Conceptual modeling approaches [30] are used for knowledge representation and maintenance of a designed system by specifying the system’s concepts and relations. Conceptual models are utilized to detect and correct errors in the early stage of system development or investigation. These approaches are usually static. They are designed to represent knowledge in a way that is humanly comprehensible. Our hybrid approach to supporting biologists’ mental working models managing combines conceptual and dynamic aspects. As we show, this aspect combination is valuable, since the conceptual component represents the various qualitative aspects of the biological mechanisms at the system level, while the executable element enables pinpointing inconsistencies, new insights, querying, and hypotheses generating and testing.

The emerging ISO 19450 standard Object-Process Methodology (OPM) [4] is a conceptual modeling approach that has originated from the information systems and systems engineering domains. OPM has been shown [31] to be significantly better in specification quality and human comprehension, compared with OMT, the main predecessor of Unified Modeling Language (UML), which is the Object Management Group’s software systems analysis and design industrial de facto standard. Recently, formal operational semantics [5, 6] and a software environment [7], as well as adaptations for modeling molecular biology systems [3], have been developed for the execution of biological OPM models. In this work we use OPM for modeling the mRNA decay and nuclear import cellular subsystem. As we show, this executable model provides a basis for generating and testing hypotheses.

To model complex systems in general and molecular biology systems in particular, OPM has inherent, built-in mechanisms for modeling biological processes, molecular functions, biological objects (e.g., molecules), object attributes, object states, object hierarchies and transient molecular structures that can be changed in a timely manner. The molecular structures and the processes that transform them can be represented at various levels of detail. The graphical language is translated on the fly into a set of natural English sentences that can be comprehended by non-expert users. To create the model presented in this work we used OPCAT [7], a freely available OPM-supporting software tool (http://esml.iem.technion.ac.il/current-projects/conceptual-systems-biology). Additional valuable feature of such a knowledge-based OPM model is its link to related research papers. OPCAT enables connecting each biological concept, be it an object, such as a specific protein, or a process, such as a catalytic reaction in which that protein is involved, to the URL of its related research paper. That paper can be easily accessed and inspected by clicking the process icon in the diagram where it appears. These characteristics make OPM highly instrumental in supporting the biologist engaged in representing and managing a mental working model of the biological system being researched.

Our conceptual model-based systems biology framework includes a set of methodological guidelines and modeling templates that help the biologist to (1) incorporate findings into the existing model, thereby augmenting and evolving it, making sure it is still executable and consistent with the known knowledge, (2) identify potential knowledge gaps and contradictions within the augmented model, and (3) if a knowledge gap is discovered, generate one or more hypotheses, incorporate them into the model, and test the model before, or even instead of, carrying out wet lab experiments aimed to close this gap.

The expert-verified (“ground truth”) model with all the relevant known mechanistic data is augmented to contain also the hypotheses or conjectures. The outcomes of executing this model are compared to the experimental findings. If no knowledge gap is discovered as a result of executing the augmented model, the conjectures that have been added can potentially become part of the new, augmented ground truth model. The model evolves over time by repeating this process while making sure that only verified facts are incorporated into it at each such iteration.

### 1.1 Object-Process Methodology (OPM)

OPM [4] is a formal yet intuitive graphical conceptual modeling language for representation, research, and development of complex systems. OPM is founded upon two elementary building blocks. These are (1) stateful objects–things that exist, such as molecules, which represent the system’s structure, and (2) processes–things that happen to objects and transform them. Processes transform the system’s objects by creating them, consuming them, or changing their states. Object states may serve as preconditions for execution of processes linked to these states.

A unique important feature of an OPM model is that it is bimodal [7]: The graphical representation–the hierarchical set of Object-Process Diagrams, OPDs–is automatically translated on the fly into Object-Process Language (OPL), a subset of natural English sentences that reflect textually all the details represented in the graphical model. The textual representation can ease the comprehension of the model by non-expert viewers who can consult the graphic and textual modalities in tandem, catering to both visual- and verbal-oriented thinkers. The opposite translation of natural English sentences to graphics is also possible.

In our first work [27] we used OPM for static knowledge representation of mRNA lifecycle, during which a knowledge gap was found manually. Recently, for executing the OPM diagrams, formal operational semantics translating OPM model into a state transition system has been defined [5, 6] and an execution environment was developed [8]. In our second paper [3], we presented OPM executable modeling templates that cater to molecular biology objects and processes–bio-OPM. We introduced adaptations to enable dynamic execution and representation of molecular biology systems and their evaluation on the transcription case study.

OPM is supported by OPCAT [8], a freely available [13] software environment, used in this work to model and execute the mRNA decay system. OPM model execution is synchronous, concurrent, and the time used in the model is discrete. During model execution, each existing object and its current state, and each process being executed are visually highlighted.

In this work we represent a major leap in our ability to utilize the system as a valuable tool for biological research. We employ the executable feature of our developed bio-OPM as a new approach for managing biological knowledge by representing conceptual mental models formally and explicitly as well as generating predictions and evaluating hypotheses. We use bio-OPM to create and manage a working model of a real investigated biological system–the mRNA decay system. By iteratively evolving the model *in silico* by adding hypothesized model facts, comparing it to *in vivo* experimental outcomes and analyzing the outcomes, we end up with a model that is consistent with respect to all our experimental findings and those in the literature.

### 1.2 mRNA Decay: Biological Background

Gene expression is a complex process, necessitating many distinct yet coupled stages. This coupling leads to coordinated regulation of RNA levels, as well as to efficient and precise protein production. The cytoplasmic levels of protein coding messenger RNAs (mRNAs) are determined by the balance between mRNA synthesis (transcription) and decay. mRNA decay [9] is an essential and well-controlled component of the gene expression process. In eukaryotes, there are two major decay pathways. One degrades the mRNAs from 5′ to 3′ by a multifactorial complex that contains the exonuclease Xrn1p and the other from 3′ to 5′ by the multifactorial complex exosome. In budding yeast, the 5′ to 3′ decay is the major mRNA decay pathway [9, 10]. We refer to the 5′ to 3′ decay machinery collectively as “decaysome” and to its separate components as “decay factors” (DFs). mRNA decay pathways have been reviewed recently [9]. Both decay pathways start with shortening of the poly(A) tail (deadenylation). In yeast, deadenylation is operated by two different protein complexes: Pan2p/Pan3p and Ccr4-NOT. Shortening of the poly(A) tail to 10–12 adenine bases or less serves as a signal for removal of the cap that protects the 5′ end by Dcp1/Dcp2. Following the decapping, the unprotected RNA is degraded exonucleolytically by Xrn1p.

**Figure 1:**
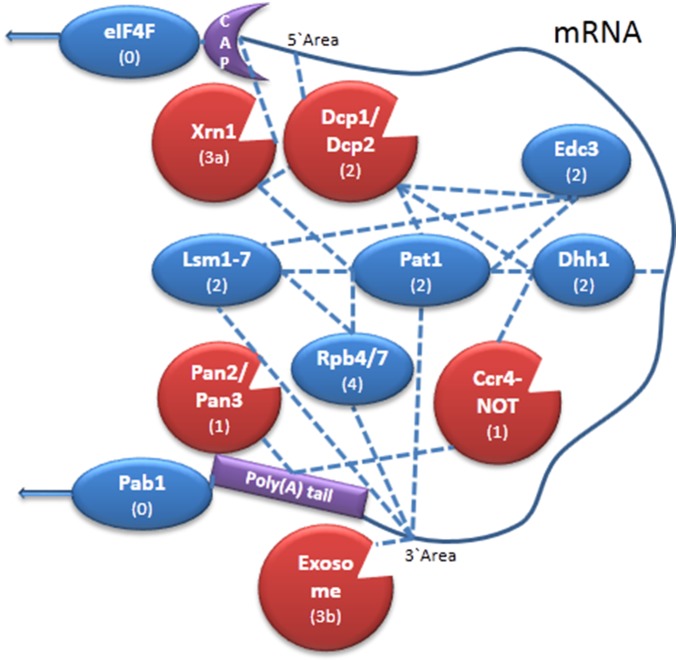
Schematic illustration of the cytoplasmic mRNA decay complex. Decaysome internal associations and associations to the mRNA, which are used in our model, are depicted with broken lines. The process in which each factor participates is as follows (see parenthesized numbers): (0) – translation, (1) – deadenylation, (2) – decapping, (3a) –5′ to 3′ degradation, (3b) –3′ to 5′ degradation, (4) coordinator.

mRNA decay is often linked to the process of translation. During translation, the cap is bound and protected by the translation initiation eIF4F complex. Therefore, in order to remove the cap, Dcp1/Dcp2 needs to outcompete eIF4F and displace it. eIF4F binding to the cap is stabilized by its interaction with the poly(A) binding protein (Pab1p), which associates with the poly(A) tail. Therefore, shortening of the poly(A) tail destabilizes the association of eIF4F with the cap structure, making the cap more accessible to the decapping complex. Several proteins regulate decapping; Edc1p, Edc2p and Edc3p are both decapping enhancers, but for simplicity, only Edc3p is included in our model. Pat1p is recruited to the mRNA while it is still being translated, and it recruits the Lsm1-7 heptamer after deadenylation [11]. The Lsm1-7-Pat1p complex is thought to stimulate the decapping step by interacting with both the oligoadenylated mRNA and the decapping complex [11]. Dhh1p is a helicase required for efficient decapping. Rpb4 and Rpb7 are subunits of RNA polymerase II (Pol II) which form a heterodimer (termed Rpb4/7). This heterodimer detaches from Pol II during transcription, binds the mRNA and escorts and regulates all post-transcriptional events. It is thought that Rpb4/7 regulates mRNA decay through its direct interaction with the Lsm1-7-Pat1p complex [12]. Figure 1 is a schematic illustration of the decaysome complex with its inter-associations, used in this work, and role of each decay factor.

The sequence of events in recruiting DFs onto the degraded mRNA described above has been extensively studied. Conventional wisdom holds that following the completion of the degradation of a certain mRNA, the decaysome reenters the decay process of yet another mRNA in the cytoplasm. Recently, however, we have discovered that following mRNA decay, the decaysome is imported into the nucleus, where it directly affects transcription [10].

Here we used the Conceptual Model-based Systems Biology OPM framework developed in [3] and the OPCAT execution environment [7, 8] for modeling and executing the mRNA decay and nuclear import subsystem. We show how this framework supports the researcher by constructing a working executable model and verifying it by reproducing experimental findings taken from 43 research papers. We also show how incomplete data is managed by attempting to fill knowledge gaps with related conjectures that can be verified or refuted. We incorporated the conjectures and refined the model iteratively till it became a working executable model with which we could reproduce the desired experimental outcomes. Whenever the experimental outcomes contradicted our *in-silico* outcomes, the conjectured mechanism was refined until both outcomes–the conceptual model and the wet lab experiment–were in agreement with each other. We used the model to evaluate possible conjectures and predict new outcomes.

## Results

### 2.1 mRNA Decay Model

#### Model Construction

The mRNA decay model presented here is a comprehensive representation of the main observations reported in 43 seminal papers, which are listed in Table S1 in File S1. In addition, our working model is based on 19 related conjectures (see Table S2 in File S1); all related to the knowledge gaps detected during model construction and execution. The model includes the common proposed mechanistic model that the biological community views as current, along with our adjustments and recent findings. All the quantitative experimental outcomes were mapped into qualitative observations (as explained in the Materials and Methods section). Being qualitative, the observations involve mechanisms and behavior, and do not involve concentrations, quantities, or probabilities. It is worth noting that the mapping for Xrn1^R101G^ (see Table 1) posed a slight dilemma, because it’s less efficient import can be mapped qualitatively into import or into non import. Our model execution, which specifies that Xrn1^R101G^ stays bound to Dcp1/2 and is released from the RNA by the compensating operation of the exosome and thus imports, led to the decision that Xrn1^R101G^ import = TRUE. This example demonstrated the utility of our model for supporting the researcher in a coherent mapping of the probabilistic experimental results into mechanistic qualitative results.

**Table 1:** Summary of *in vivo* observations regarding Xrn1p and Dcp2p import dependencies.

	Deletion Type	Xrn1p Import	Dcp2p Import
1	WT	**+**	**+**
2	XRN1 deletion	N/A	**+** Dcp2p is imported into the nucleus as efficiently as in WT cells.
3	Xrn1 5′end RNAbinding site mutant(R101G)	**+** Xrn1^R101G^ is imported tothe nucleus less efficientlythan in WT cells.	**+** Dcp2p is imported into the nucleus as efficiently as in WT cells.
4	Xrn1 active sitemutant (D208A)	− Xrn1^D208A^ is not imported into the nucleus.	**–** Dcp2p is not imported into the nucleus.

Our mRNA decay model comprises 152 objects and 110 processes, of which 62 are leaf processes, i.e., basic non-decomposed processes that were not further in-zoomed. Each basic process in our model is a molecular function classified as (1) catalyzing, (2) binding/dissociation, or (3) transporting [3]. The remaining 48 processes are at higher levels, and they are comprised directly or indirectly of these three types of molecular functions.

**Figure 2:**
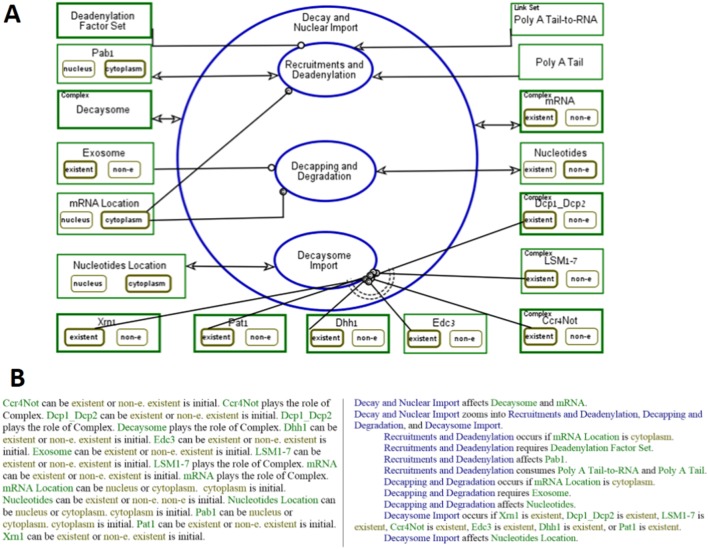
mRNA Decay and Nuclear Import Process. (A) **The mRNA Decay and Nuclear Import** Process is in-zoomed into three sub-processes; **Recruitments and Deadenylation, Decapping and Degradation** and **Decaysome** **Import**. (B) The corresponding, automatically-generated textual Object-Process Language (OPL) paragraph that reflects textually what the diagram represents graphically.

Figure 2 is an OPD in which **Decay and Nuclear Import**–the initial top-most process in the mRNA decay model–is zoomed into, exposing three subprocesses: **Recruitments and** **Deadenylation**, **Decapping and Degradation** and **Decaysome Import**, which are executed serially, as indicated by their top-to-bottom spatial ordering within the in-zoomed process. The first process, **Recruitments and** **Deadenylation** process, changes the states of several objects. For example, to model the fact that **Recruitments and** **Deadenylation** process consumes the **poly-A tail** (depicted near the right top in Figure 2), we use a consumption link. The consumption link is an arrow emanating from the **Poly A Tail** object to the **Recruitments and** **Deadenylation** process. Similarly, the **Recruitments and** **Deadenylation** process consumes also the **Poly A Tail-to-RNA Link Set**, meaning that the poly A tail is dissociated from the RNA.

The presence of the object **Deadenylation Factor Set** and the fact that the object **Poly A Tail** is existent and **mRNA Location** is at the **cytoplasm** state are three of the requirements comprising the precondition for the execution of the **Recruitments and** **Deadenylation** process. The requirement that **mRNA** be at the **cytoplasm** state is expressed by the condition link–a line ending with a small circle and a “c” inside it, connecting the state **cytoplasm** of the **mRNA** **Location** object (depicted near the left bottom in Figure 2) to the **Recruitments and** **Deadenylation** process. If this condition is not satisfied, **Recruitments and** **Deadenylation** process is skipped (deactivated), and the next process, **Decapping and Degradation**, is triggered.

**Deadenylation Factor Set** is connected to the **Recruitments and** **Deadenylation** process with an instrument link, a line ending with a small circle at the process end, specifying that **Deadenylation Factor Set** is required for **Recruitments and** **Deadenylation** to happen. **T**he object **Deadenylation Factor Set** is a composition of the factors executing **Recruitments and** **Deadenylation**, which are the objects **Ccr4-Not** and **Pan2–Pan3 Complex**. These are exposed and used in the lower-level, refined diagrams, which is not shown.

The state of the object **Decaysome** changes during each process of the **Decay and Nuclear Import** process, as expressed by the effect link, depicted as a bidirectional arrowhead between this object and the **Decay and Nuclear Import** process. The details of these changes are shown in the OPDs, in which **Decay and Nuclear Import** is in-zoomed (not presented). The entire combined model of the mRNA decay, import and transcription can be downloaded from [13] and execution records appear in File S2.

#### Model Evaluation

After constructing the OPM decay model, we tested it in the following manner. We executed the model under each of the initial conditions corresponding to the experiments described in Table S1 in File S1. We then verified that the outcomes (see columns 5 and 6 of Table S1 in File S1) match. Inconsistencies between the results from the execution of the computational OPM model and the biological experiments were used to reevaluate and adjust the computational model. Since much of the mechanistic data in the mRNA decay process is still unknown, the adjustments also included the addition of conjectures, as explained below. This process of gradual refinement and incorporation of additional conjectures into the model was iterated until the executions were in line with the wet-lab experimental outcomes.

We used the verified model to generate predictions, evaluate hypotheses and test experimentally part of them, as presented in the following sections.

### 2.2 Conjectures Incorporation

Recently, we found that mRNA decay factors, normally detected in the cytoplasm, shuttle back and forth between the cytoplasm and the nucleus [10]. Mutational analysis suggested that nuclear import of Xrn1p and Dcp2p, and possibly other DFs, requires complete degradation of the mRNA [10]. However, the current published data was insufficient for creating an executable model. To overcome this limitation we made plausible conjectures, which are presented in Table S2 in File S1. In total, 19 assumptions and conjectures were incorporated into the model this way, many of them expressing temporal aspects. Several conjectures were related to the unknown mechanism of decay factors import into the nucleus. These included two major questions: (1) What are the prerequisite conditions for DFs import? Specifically, do the DFs remain bound to the mRNA until it is completely digested? (2) What are the protein interactions and the sequence of events involved in the import of each DF? Does each DF import independently or together with other DFs in a dependent manner?

We incorporated into the model our conjectured mechanisms, related to these questions, and tested them on the model to match all known experiments (see Table S1 in File S1). First, we incorporated into the model the conjecture that the DFs import after they are released from their direct or indirect association to the mRNA by an exonuclease (either Xrn1p or the exosome) which degrades the mRNA. Inserting this conjecture allowed for a logical sequence of events by which the import of DFs to the nucleus begins only after the entire process of mRNA degradation is completed [10]. We later confirmed experimentally this conjecture as described below. Second, the specific interactions occurring within the decaysome complex before its import into the nucleus are not known and thus had to be investigated. To better understand these possible sub-complexes within the decaysome complex, we tested several hypotheses on the model. Initially, we incorporated a simplifying assumption that each DF is imported independently into the nucleus, accompanied by a distinct import factor (e.g., karyopherin). Next, we incorporated a variation for the import timing of the decapping complex, Dcp1/2. The common working models suggests that Dcp1/2 is recruited before decapping and dissociates from the mRNA after it executes decapping [9, 16]; thus, it may import immediately thereafter, even before the RNA is degraded. Third, we inserted Roy Parker’s suggestion [9] for the DFs structure that two major complexes assembled on mRNAs before decapping; the Pat1p-Lsm1-7-Xrn1p complex and the Dcp1/2-Edc3p-Scd6p-Dhh1p complex.

In order to evaluate the feasibility of these conjectured mechanisms, we incorporated each conjecture into the model and evaluated how the model execution outcomes match our experimental results (see Table 1 and Table S1 in File S1). These models executions failed to reproduce the experimental outcomes for the *Xrn1^D208A^* mutation (Table 2 columns 1, 3), which showed dependency of Dcp2p import on the nuclear import efficiency of Xrn1p [10]. Table 2, column 1, lists the contradicting *in-silico* results, regarding Xrn1p and Dcp2p import, for the conjecture that each DF is imported independently into the nucleus. Roy Parker’s suggestion (Table 2, column 3) was not in-line with our experimental results, which implies that the proposed Decaysome structure is change after decapping.

**Table 2:** *In-silico* DFs import results for permutations on DFs interactions, tested for *Xrn1^D208A^* mutation.

		Permutation No.										
		1	2	3	4	5	6	7	8	9	10	11
**Model Initiation: Associating Factors** **(association** **yes/no = 1/0)**	**Xrn1-Dcp1/2**	0	1	0	1	1	1	0	1	1	1	1
	**Xrn1-Pat1**	0	0	1	1	1	1	1	1	1	1	1
	**Dcp1/2-Edc3**	0	0	1	0	0	0	0	1	1	1	1
	**Dcp1/2-Dhh1**	0	0	0	0	0	1	0	1	1	1	1
	**Dcp1/2-Pat1**	0	0	0	1	1	1	1	1	1	1	1
	**Lsm1-Edc3**	0	0	0	1	1	1	1	1	1	1	1
	**Lsm1-Pat1**	0	0	1	0	0	0	0	0	0	0	0
	**Lsm1-Rpb4/7**	0	0	1	1	1	1	0	1	0	1	0
	**Pat1-Edc3**	0	0	0	0	0	0	0	0	0	0	0
	**Pat1-Dhh1**	0	0	0	0	0	1	0	1	1	1	1
	**Pat1-Rpb4/7**	0	0	1	0	1	0	0	1	0	0	1
	**Ccr4Not-Dhh1**	0	0	1	1	1	1	1	1	1	1	1
**Model Predictions:** **Decay Factors** **Import (yes/no = +/−)**	**Xrn1**	−	−	−	−	−	−	−	−	−	−	−
	**Dcp1/2**	*+	−	*+	−	−	−	−	−	−	−	−
	**Edc3**	+	+	+	+	*−	+	+	*−	*−	*−	*−
	**Lsm1**	+	+	*−	+	*−	+	+	*−	*−	*−	*−
	**Pat1**	*+	*+	−	−	−	−	−	−	−	−	−
	**Dhh1**	+	+	+	+	+	−	+	−	−	−	−
	**Rpb4/7**	+	+	−	+	−	+	+	−	+	−	−
	**Ccr4Not**	+	+	+	+	+	−	+	−	−	−	−

The *Xrn1^D208A^* mutation forces the factors that directly or indirectly interact with *Xrn1^D208A^* to stay in the cytoplasm and not import into the nucleus. Using this mutation, we initiated our model with various permutations on decay factors interactions, executed the model for each such permutation to predict the import status of each decay factor and compared it to our *in-vivo* observations.

There are 4096 (212) possible permutations for the interactions of the decay factors. We chose to execute 11 permutations (columns 1–11), where 3 of them (column 4, 6, 7) were verified by the model to match the experimental findings. Two of these options are presented graphically in Figure S2 in File S1. The executions in column 1, 2 and 3 includes our initial conjectures and do not match the experimental findings. In the executions presented in columns 4–11, the direct interactions between Pat1 and Edc3, and Lsm1-7 were eliminated, since our experiments argued against them (see Figure 4). The “Model Initiation” rows represent the model initiations with various possible decay factors interactions and “Model Predictions” rows represent the model outcomes of decay factors import status. The (*) sign in the “Model Predictions” rows highlights the *in-silico* outcomes that contradict our in-*vivo observations presented in* *Figure 4* *and* *Table 1* *(i.e., for Xrn1^D208A^ we observed* that Xrn1 = no import, Dcp1 = no import, Dcp2 = no import, Pat1 = no import, Lsm1 = import and Edc3 = import).

We then refined the model with a different conjectured mechanism. We propose that Dcp2p form physical interactions with Xrn1p during its import, based on our observation [10] that Xrn1p import is required for the import of Dcp2p. Thus we modeled that Xrn1p and Dcp1/2 import as a single complex, while the other DFs import independently. Xrn1p was modeled to associate with the 5′end, where it is stabilized by its associations to Dcp1/2 prior to the nuclear import. This conjectured mechanism fits into the model context, complies with all the published data (see Table S1 in File S1) that the model is based on, and yields the known experimental outcome, as presented in Table 3 line 9. We conducted further wet lab experiments to determine which other associations (except for the Xrn1-Dcp1/2 association) may exist during the import (see below) and later refined this conjecture.

**Table 3:** *In-silico* DFs import results and processes activation when deleting one factor/domain at a time.

Model Initiation		Model Predictions:Decay Factors Import(yes/no = +/−)									Effect On ProcessesActivation	
Deletion:		Dhh1	Ccr4NOT	Lsm1-7	Dcp2	Dcp1	Edc3	Xrn1	Pat1	Number of lowest level processes that failed to execute (%)	Main Processes thatexecuted successfully	Main processes that fail toexecute (Deadenylation/eIF4FDissociation/Pab1Dissociation/Decapping/5′ to 3′Degradation)
**WT**	**1**	+OP	+OP	+OP	+OP	+OP	+OP	+OP	+OP	13 (20.9%)	Deadenylation, Pab1Dissociation,eIF4FDissociation, Decapping,5′ to 3′ Degradation	3′ to 5′ degradation
Δ***pat1***	**2**	+P	+P	−P	−P	−P	+P	+P	NA	40 (64.5%)	Deadenylation, Pab1Dissociation, eIF4FDissociation, 3′ to 5′degradation	Decapping, 5′ to 3′ degradation
Δ***xrn1***	**3**	+P	+P	+P	+OP	+P	+P	NA	+P	17 (27.4%)	3′ to 5′ degradation	5′ to 3′ degradation
Δ***edc3***	**4**	+P	+P	+P	+P	+P	NA	+P	+P	17 (27.4%)	Deadenylation, Pab1Dissociation,eIF4FDissociation, Decapping,5′ to 3′ Degradation	3′ to 5′ degradation
Δ***dcp1 and Δdcp2***	**5**	+P	+P	+P	NA	NA	+P	+P	+P	27 (43.5%)	Deadenylation, Pab1Dissociation, 3′ to 5′degradation	eIF4F Dissociation, Decapping, 5′ to 3′ degradation
Δ***lsm1***	**6**	+P	+P	NA	−P	−P	+P	+P	+P	32 (51.6%)	Deadenylation, Pab1Dissociation,eIF4FDissociation, 3′ to 5′Degradation	Decapping, 5′ to 3′ degradation
Δ***ccr4-***	**7**	+P	NA	+P	+P	+P	+P	+P	+P	15 (24.1%)	Deadenylation, Pab1Dissociation,eIF4FDissociation, Decapping,5′ to 3′ Degradation	3′ to 5′ degradation
Δ***dhh1***	**8**	NA	+P	+P	+P	+P	+P	+P	+P	26 (41.9%)	Deadenylation, Pab1Dissociation, eIF4FDissociation, 3′ to 5′Degradation	Decapping, 5′ to 3′ Degradation
**Xrn1 Active Site^−^ (** ***xrn1^D208A^*** **)**	**9**	+P	+P	+P	−OP	−P	+P	−OP	+P	17 (27.4%)	Deadenylation,Pab1Dissociation, eIF4FDissociation, Decapping,3′ to 5′ Degradation	5′ to 3′ Degradation
**Xrn1 5′RNA Binding Site^−^ (** ***xrn1^R101G^*** **)**	**10**	+P	+P	+P	+OP	+P	+P	+OP	+P	11 (17.7%)	Deadenylation, Pab1Dissociation, eIF4FDissociation, Decapping,3′ to 5′ Degradation	5′ to 3′ Degradation

“+” represent import and “−” represent not-imported. “O” represents actual observation from experiments (see [10]), and “P” represents *in-silico* predictions that may not be experimentally tested. NA – not applicable.

### 2.3 Predictions Generation

One advantage that our computational model provides is its capacity to pursue *in silico* experiments and obtain results that can later be examined experimentally.

We used our verified computational mRNA decay model to predict the effect(s) of deleting some decay factors (or domains/site) on the import of other decay factors and on processes activation. As a case in point, we chose 10 permutations - deletions of molecule/domain - and executed the model. The major processes and lowest level processes that fail to execute are summarized in Table 3. In our models, major processes are further in-zoomed into molecular functions which are the basic, lowest level system processes, so they cannot be further in-zoomed.

Our model predicts new possible results. One result is related to *PAT1* deletion (see Table 3 line 2). In this case, the execution predicts that 3 decay factors, Lsm1-7, Dcp1p, and Dcp2p, will not import into the nucleus. Analyzing the execution reveals that Pat1p deletion results in the following scenario:

(1) Lsm1-7 is not recruited to the mRNA and is therefore not imported into the nucleus. This was based on our conjecture that RNA binding is a prerequisite for import.

(2) Dcp1/2 complex is not recruited to the mRNA by Lsm1-7-Pat1p and does not bind any of the factors that normally bind it, Edc3p, Dhh1p, and Pat1p; it cannot execute decapping and is not imported into the nucleus.

(3) The alternative 3′ to 5′ degradation pathway is executed by the exosome.

When analyzing the deactivated processes in this scenario, we found that 40 out of 62 (64.5%) downstream (lowest level) processes were not activated when *PAT1* was deleted. This analysis suggests that Pat1p is a major decay coordinator, as its deletion impacts many of the decay mechanisms and nuclear import. This *in-silico* based conclusion is supported by the observations that Pat1p is a critical component of the major mRNA decay pathway due to its role as an inhibitor of translation initiation as well as its role as scaffold of the major mRNA decay complex [17]. We also predicted that *LSM1* Deletion will affect the import of Dcp1 and Dcp2 and the activation of 51.6% of the processes. The effect of *PAT1* and *LSM1* deletion on the import of other decay factors needs to be further validated experimentally.

In addition, our model predicts that for the *Xrn1* ^D208A^ mutation, which ‘stuck’ the *xrn1* ^D208A^ mutant strain with its interacting partners on the RNA, all decay factors except for Dcp1/2, will import (see Table 3, line 9). We note that this prediction was based on our simplified conjecture that the DFs import is independent, since the mechanism was still unknown. Dcp1/2 is not imported since it was conjectured to be connected to Xrn1p. Since our group was interested in investigating this mostly unknown DFs structure during import, we decided to test experimentally this prediction. In the following section, we show the experimental evaluation of this prediction and our model utilization for refining our initial conjecture.

### 2.4 Experimental Validation of Predictions and *In-silico* Evaluation of Hypotheses

We tested experimentally two conjectures. We then used our model to evaluate the validity of possible explanations to our observations.

**Figure 3:**
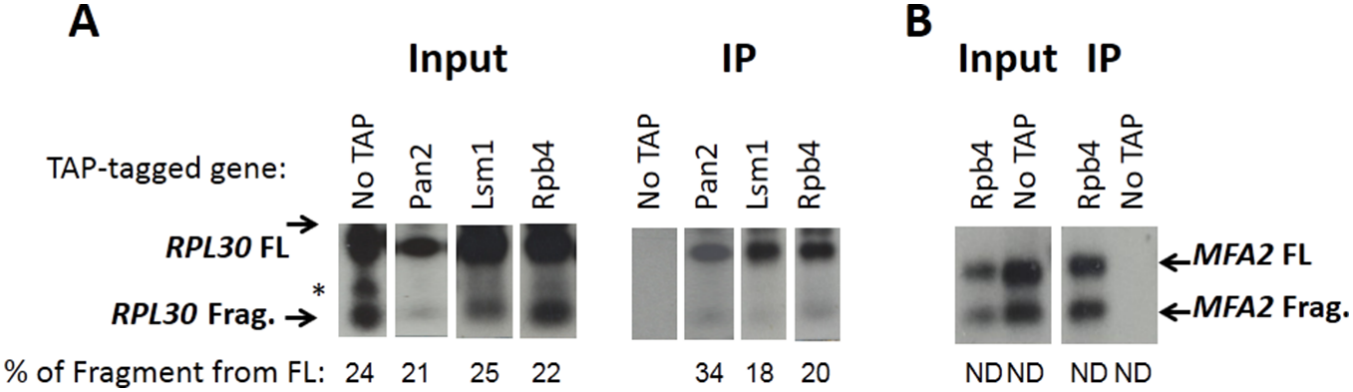
Experimental validation of our model-based hypothesis shows that some mRNA decay factors are associated with both full length mRNA and its degradation intermediate. (**A**) Cells expressing *RPL30pG* (“Construct B” in [****18]), whose 3′ non-coding region contains (G)18 tract, and carrying the indicated TAP-tag in place of the natural genes were used. The indicated TAP-tagged proteins were affinity purified using two affinity steps as described previously [****19], under conditions that minimized RNA degradation *in vitro*. The RNA was extracted and subjected to Northern analysis [****24]. The probe contained the (C)18 and flanking regions; it was hybridized under stringent conditions (75°C) and, except for one non-specific band (marked by asterisk), was highly specific [****18]. Accumulation of the degradation fragment (denoted “Frag.”) was due to the (G)18 tract that blocked the 5′ to 3′ exonuclease activity of Xrn1p [****20, ****21]. The Fragment therefore spans from the (G)18 tract till the 3′ end of the RNA (88 b including the G tract). Input was obtained by subjecting total RNA (before IP) to Northern analysis; IP are the affinity purified samples. Percent of “Fragment” from FL was determined by measuring radioactivity using the PhosphImager technology. All lanes were taken from the same gel. (**B**) Cells expressing *MFA2pG*, whose 3′ non-coding region contains (G)18 tract [****20, ****21], and carrying *RPB4*-TAP in place of *RPB4*, or carrying untagged *RPB4* (“No TAP”) were used. TAP purification followed by Northern analysis was done as in A. The affinity purification of Rpb4-TAP was performed three times (twice for examining *RPL30pG* [A] and once for examining *MFA2pG* [B]), and those of Pan2 and Lsm1 repeated twice. Whereas the % of “Fragment” from full length mRNA was different in different experiments (it is highly sensitive to the strain background and to the nature of the mRNA, and somewhat also to the proliferation conditions), in all cases, the proportion of Fragment in the immunoprecipitated material was similar to that in the input material, as shown in this figure. FL – full length. Frag. – “Fragment”. ND = not determined.

To test the conjecture that DFs remain bound to the mRNA until it is completely digested, we performed RNA immune-precipitation (RIP) assay to examine whether some DFs associate with partially digested mRNAs. We used a previously published construct, in which a poly-G sequence ((G)_18_) is inserted into either the *RPL30* or *MFA2* 3′ untranslated region (UTR). Since a (G)_18_ tract serves as a barrier to exonuclease activity [20, 21], 5′ to 3′ mRNA degradation generates a degradation intermediate fragment that stretches from the (G)_18_ tract to the 3′ end (called “Fragment”). Figure 3 shows that some DFs associate with the full length *RPL30* and *MFA2* mRNAs, as well as with the corresponding “Fragment”. Reproducibly, the proportion of Fragment in the immunoprecipitated material (the IP lanes in Figure 3) was similar to that in the input material. Interestingly, even Pan2, whose known function is to degrade the poly(A) tail [9], remains bound to the decaying RNA even after decapping and degradation of the entire open reading frame and a portion of the 3′ untranslated region. Ccr4, another component of the deadenylation complex [9], remains bound to Fragment as well and does not leave the complex following deadenylation (results not shown). These results are consistent with our conjecture that these factors remain bound to the 3′ portion of the RNA during the 5′ to 3′ degradation of most of the RNA. Thus, the DFs we tested seem to stay attached to the mRNA after deadenylation and decapping, and after 5′ to 3′ exonucleolytic degradation has begun. These results are not trivial, because at least Pan2p and Ccr4p, components of the deadenylation process, are expected to leave the complex after deadenylation is completed. These results support our modeled conjecture, at least regarding factors that associate with the 3′-UTR. This conjecture is also supported by previous findings that the Lsm1-7p complex remains associated with the 3′ UTR of the mRNP after decapping and during the exonucleolysis by Xrn1p [11].

Next, we tested experimentally the predictions we had made regarding the dependencies between the decay factors during import. Our predictions were based on our initial conjecture that the decay factors are not associated during import, except for Xrn1p and Dcp1/2 which were suggested to be associated [10]. To examine which interactions exist between the DFs before and during their import into the nucleus, we used the *xrn1* ^D208A^ mutant strain. This enzyme dead mutant form is capable of binding the RNA 5′end, but not degrading it. As a consequence, *Xrn1* ^D208A^ is left “stuck” in the cytoplasm, in complex with the 5′ end of the RNA that cannot be degraded, along with all its interacting partners [10]. By determining which factors stayed “stuck” with *Xrn1* ^D208A^ and could not import in *Xrn1* ^D208A^ mutant cells, we were able to analyze the possible interactions that existed between the decay factors during import (see Table 2).

**Figure 4:**
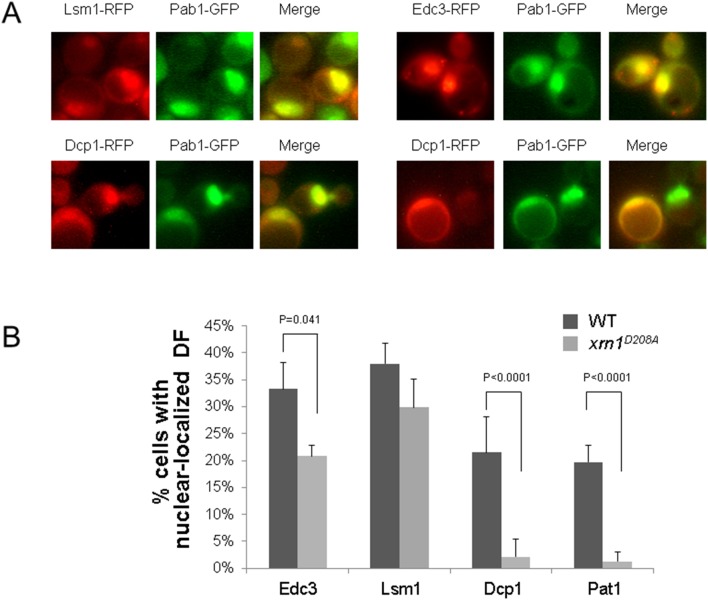
Experimental validation of model’s predictions shows that import of some decay factors is independent of Xrn1p exonuclease activity. XRN1 (WT) PAB1-GFP xpo1-1, mex67-5 cells, or xpo1-1, mex67-5, Δxrn1 cells expressing xrn1D208A-GFP and RFP fusion of the indicated DFs were proliferated at 24°C and then shifted to 37°C for 1 h; images were taken as previously described [****10]. (A) Representative images of WT cells expressing the indicated proteins after 1h incubation at 37°C. Pab1-GFP, whose export is dependent on Xpo1p and Mex67p, serves as a nuclear marker, as described in [10]. Arrows point at examples of nuclei carrying both fluorescent proteins. All factors were cytoplasmic at 24°C ([****10] and not shown) (B) Percentage of cells with nuclear localization of the indicated DF was determined, as described previously [****10]. Mean values ± SD are shown. P-values of any pairwise difference that was <0.05 is indicated.

We employed a nuclear import assay for analyzing import of four factors. The assay involves a strain carrying specific mutations in *XPO1*, and *MEX67*, which cause a block in protein export upon shifting to the non-permissive temperature (37°C) [10]. The strain also expresses Xrn1^D208A^, instead of Xrn1p, which cannot import [10]. Figure 4 shows that, despite their capacity to shuttling efficiently in WT cells [10], Pat1p and Dcp1p failed to import in this *xrn1^D208A^* mutant strain. We interpreted the failure of Dcp1p and Pat1p to import as an indication of their physical interaction with Xrn1^D208A^. Efficient import of Lsm1p, import of Edc3p and failure of Dcp1p to import are in accord with model predictions. However, the non-import of Pat1p contradicted predictions of the model. We note that Edc3p import was compromised but not blocked. Taken together, these results indicate that some DFs can be imported independently of Xrn1p import; these results further suggest that import of some DFs, i.e. Dcp1p, Dcp2p and Pat1p, is dependent on Xrn1p import. We propose that import of DFs is more complex than our original conjecture. Since Dcp1p, Dcp2p, and Pat1p import seems to be dependent on Xrn1p, the simplest model posits that the four DFs (Xrn1p, Dcp1p, Dcp2p, and Pat1p) import as a complex, while Lsm1p and Edc3p are imported either independently (Lsm1p probably in the context of Lsm1-7 complex) or in complex with other DFs. Thus, a portion of the Decaysome can be released from the degraded mRNA when *Xrn1* ^D208A^ active site remains bound to the RNA in the cytoplasm. These results prompted us to change the conjecture we used in the model. We refined our model with a new module and used it to evaluate other possible interactions that are consistent with our observations. Since there are 4096 possibilities for interactions between the decay factors (there are 12 different links between the depicted DFs, shown in Figure 1, and two possible states, existence: yes/no, for each link, hence 2^12^), a computational approach is required to analyze the appropriate options. Figure S2 in File S1 presents graphically two possible conjectures that match our experimental results. Table 2 columns 4–11 presents additional possibilities (i.e., different permutations on interaction-related dependencies) that were executed on our model. Columns 4, 6, and 7 present conjectures that match our experimental results. The complete execution and analysis of all 4096 options is laborious and requires programing a sub-routine to initiate and run all the permutations. Hence it is outside the scope of this paper. Nevertheless, the *in-silico* executions we show here further exemplify the computational predictive power of the approach for evaluating the validity of conjectures.

During import, one of the possibilities (see Table 2 column 4, Figure S2 (A) in File S1) posits that the decaysome is divided into the following three complexes: (1) Xrn1p-Dcp1/2-Pat1p, (2) Rpb4/7-Lsm1-7-Edc3p, and (3) Ccr4Not-Dhh1p, which may import as one complex or within various sub-complexes. Another option suggests the following two complexes (Table 2 column 6, Figure S2 (B) in File S1): one complex consists of Dcp1/2-Xrn1p- Pat1p-Dhh1-Ccr4-Not and the other contains Rpb4/7-Lsm1-7-Edc3p. The difference between these two options is the link between Dhh1p and Pat1p. Rpb4/7 was chosen to be separated from complex (1) because Rpb4p is the only reported case of DF that contains a functional NLS [22]. These new models successfully reproduced our experimental findings (see Table 2 columns 4, 6, 7 and Figure S1 in File S1 for snap-shot of model execution outcomes and [13] for downloading the updated model). Further experimentation is required to determine which one of the options described above holds true. For example, to narrow the possible options, we recommend an additional experiment for determining Dhh1p import under the *Xrn1* ^D208A^ mutation.

An interesting implication of our observation that Pat1p import is different than Lsm1p import is that Pat1p disengages from the Rpb4/7-Lsm1-7-Pat1p complex at some point between recruiting Lsm1-7 to the mRNA and DF import. Currently, the timing and the regulation of this step, as well as its importance, are still unknown.

## Discussion

In this paper we present a computational working model of the relatively complex and yet largely unknown mRNA decay process followed by the nuclear import of the decay factors in *S. cerevisiae*. We use OPM and bio-templates that were recently developed [3] to establish this working model of the mechanisms underlying the mRNA decay process, based on cumulative knowledge extracted from 43 research papers. We show how the conceptual model supports the biological researcher and facilitates the establishment of a coherent working model, the formulation and evaluation of biological hypotheses and generation of predictions. These predictions were experimentally validated or refuted, enabling iterative perfection of the model and its alignment with all the wet lab experimental outcomes.

Executable models [1] that enable dynamic analysis of biological system mechanisms were shown to generate valuable discoveries [1, 2]. For example, Fisher et al. [2] modeled gene expression interactions during the Vulval cell fate determination in *C. elegans* using the Statecharts formalism, and verified the mechanistic model with experimental results during which two predictions were raised and validated experimentally. Most of these executable biology efforts were aimed to test biological systems that had been thoroughly investigated, where much of the mechanistic data is known. To the best of our knowledge, the work presented here is the first executable model of the mRNA decay process. This has been a challenging task, since much of the knowledge is unknown.

Our model represents a spectrum of complexities of biological processes and their temporal order, from the lower level molecular functions to the entire mRNA decay process and consecutive nuclear import of DFs. The effect of each process on hierarchical structures such as molecules, complexes, domains and binding sites, is also modeled explicitly.

The mRNA decay model includes 110 processes, 62 lower level processes and 152 objects specifying the mechanisms underlying the mRNA decay process. To test the relevance of our model, we compared the model execution results with 43 known experimental observations. Whenever the execution did not match the experimental findings, we adjusted the model or added reasonable conjectures into it to close knowledge gaps that had been found. This process of gradual refinement and incorporating additional assumptions and conjectures into the model was iterated until the executions were in line with the wet-lab experiment outcomes. In total, 19 conjectures were incorporated into the model this way.

*In silico* testing of conjecture 10 (Table S2 in File S1) allowed us to dismiss this conjecture, since the final executable results did not match published data. A different conjecture (conjecture 11) was then conceived. Furthermore, the results presented in Figure 3 supported conjecture 15. Thus, the process of constructing the model helps to pinpoint missing information and devise conjectures as to the behavior of the system. These conjectures are later tested experimentally both *in silico* and in the test-tube. Importantly, the ability to dismiss conjectures by *in silico* experiments saves both researchers’ time and monetary resources that would have otherwise been wasted on testing wrong hypotheses. Thus, our conceptual model based approach allows the researcher to benefit from the advantage of being able to concentrate efforts on modeling conjectures about underlying biological mechanisms that go hand-in-hand with the reported wet-lab results and can be tested and verified also *in silico.*

The refined model in our research was used to investigate the effect of mutating/deleting a specific decay factor on the behavior of the entire system. Specifically, we analyzed affected processes and examined import of DFs into the nucleus. Several interesting predictions were made. Surprisingly, deletion of *PAT1* and *LSM1* had an effect on import of other decay factors, while deletion of *CCR4*, *DHH1, EDC3, DCP1* and *DCP2* did not affect the *in silico* import of other decay factors.

Xrn1 seems to represent a unique case. Deletion of *XRN1*, or inserting a mutation that compromises its ability to bind the uncapped 5′end of the mRNA, did not affect the import of any of the other factors *in silico*. This was demonstrated *in vivo* when analyzing the effect of Xrn1 mutation/deletion on Dcp2 import [10]. However, the *xrn1^D208A^* mutant, which has the ability to bind the RNA 5′end, but not to degrade the RNA, blocked the import of Dcp1/2 according to the model. We previously proposed that as long as the active site is occupied with RNA, Xrn1p import is inhibited [10]. We also proposed that since Dcp2p binds Xrn1p during RNA degradation and during subsequent import, the D208A mutation would not only prevent import of Xrn1p, but also that of Dcp2p [10]. This scenario was tested experimentally for four factors: Pat1p, Dcp1p, Edc3p and Lsm1p (Figure 4). As the model predicts, Dcp2p [10] and Dcp1p import was inhibited by this mutation and Lsm1p and to some extent also Edc3p, were imported independently of Xrn1p (Figure 4). Surprisingly, Pat1p import is inhibited by this mutation. The capacity of Lsm1p to import under conditions that Pat1p cannot import suggests that the Lsm1-7 interacts with Pat1p only transiently during the decay process, and it should dissociate from Pat1p at some point before import. Therefore, the process of release of DFs from the 3′end of the mRNA may be more complex than previously appreciated. Lsm2p interacts with the Rpb4/7 heterodimer [23, 24], which contains an NLS [22]. Thus, it is possible that Lsm1-7 import relies on its interaction with Rpb4/7. We used our extended model to evaluate several possible perturbations on the decay factors interactions, shown in Table 2, to predict possible molecular contexts relevant to their import.

Recently, Roy Parker’s group proposed [9] that two major complexes assemble on mRNAs before decapping and mediate mRNA decay. One consists of the Pat1p-Lsm1-7-Xrn1p complex and is located at the 3′end of the RNA, and the second complex contains Dcp1/2-Edc3p-Scd6p-Dhh1p and is located at the 5′end of the RNA. Our refined model of the decaysome import also suggests the existence of two distinct complexes. Based on our model analysis (Table 2 column 4 and Figure S2 (A) File S1), we propose the following two complexes: one imported complex consists of Pat1p-Dcp1/2-Xrn1p and the other contains Rpb4/7-Lsm1-7-Edc3p-Dhh1p-Ccr4-Not. We bound the Dhh1p-Ccr4-Not complex with the Rpb4/7-Lsm1-7-Edc3p complex (see Figure S2 (A) in File S1) based on the recently found additional interaction [29] (not included in our model) that Dhh1 binds either Pat1 or Edc3. Another plausible option may be the following two complexes (Table 2 column 6, Figure S2 (B) in File S1): one complex consists of Dcp1/2-Xrn1p-Pat1p-Dhh1-Ccr4-Not and the other contains Rpb4/7-Lsm1-7-Edc3p. All these options are experimentally testable, by testing the import of Dhh1p. It is possible that during the decay process, two major complexes function as Parker suggested, whereas following the degradation, the complexes are reshuffled prior to their import to the nucleus. Specifically, we have examined *in silico* the following scenario: Xrn1p, which is known to be located at the 3′RNA [25] changes its binding feature and binds the 5′RNA region before starting its catalytic activity. Pat1p, which is associated with the 3′RNA [9] and also binds Xrn1p, may mediate Xrn1p recruitment to the 5′RNA. Dcp1/2 is known to bind the 5′ region of the mRNA [28]. Decapping leads to a change in Dcp1/2 conformation, but it still remains bound to the 5′ region of the RNA. This conformational change stimulates Xrn1p recruitment to the 5′end, bringing Xrn1p active site near the decapped end of the RNA. This facilitates the entry of this end to the active site, thus enhancing the RNA degradation. Executing this scenario by our modeling system was successful and coherent with the known experimental findings that served the basis for the *in silico* system (data not shown), but no experimental validation has been performed.

*In silico* deletion of *PAT1* resulted in deactivation of 64.5% of system processes. It also resulted in lack of import of Dcp1/2 and Lsm1-7. This analysis points at Pat1 as a major decay coordinator, the deletion of which impacts many of the mechanisms of decay processes and the import of at least three DFs into the nucleus. This *in silico* based conjecture is supported by previous observations, suggesting that Pat1p is a major scaffold of the mRNA decay complex [17]. Pat1p is suggested to recruit Dcp1/2 (see Table S1 in File S1) and Lsm1-7 (see Table S2 in File S1 and [17]) to the mRNA. This may explain why these factors are not imported into the nucleus in this model execution. We postulate that import of the DFs requires prior assembly with the decaying mRNA. Consequently, our model system posits that Dcp1/2 and Lsm1-7 need to be recruited to the mRNA in order to be later imported to the nucleus. Interestingly, this *in silico* analysis suggests that nuclear import of Xrn1p, Edc3, and CCR4-Not/Dhh1p complex does not depend on Pat1p, although Pat1p interacts with Dhh1p, Xrn1p, and Edc3p during the decay process. Hence, some rearrangement of the interactions network must occur after RNA degradation terminates and before import. We also postulate that any given DF cannot import while still binding to the RNA. Releasing a DF or a complex of a number of DFs from the RNA can be accomplished by degrading the RNA either by 5′ to 3′ degradation by Xrn1p or by 3′ to 5′ degradation by the exosome, or by both exonucleases simultaneously. Obviously, in the absence of Xrn1p, only the exosome can release the DFs and permit their import.

Edc3 import shows some dependency on Xrn1. It is possible that Edc3 differentially interacts with Pat1p/Xrn1p/Dcp1/2 and Lsm1-7/Rpb4/7 complexes, depending on the substrate mRNA. For instance, Edc3 recruits Dcp1/2 to the *RPL28B* mRNA and promotes its decapping, independently of deadenylation (and therefore, presumably independently of Lsm1-7) [26].

The above results show that our Conceptual Model-based Systems Biology framework can be highly instrumental in supporting the biological researcher in understanding and analyzing a complex, yet unexplored, molecular biology systems, driving its execution, and supporting hypotheses generation and validation. Moreover, our model serves as a graphical navigable knowledge base connecting each biological process or object to its related research paper that can be inspected simply by clicking the process in the visual framework.

As we show here, our model can detect inconsistencies in biological conjectures before conducting costly and lengthy wet lab experiments. This model augmentation and validation process demonstrates that our modeling method can be used not just to examine model consistency, but also to predict the validity of conjectures *in silico* before testing them *in vivo* or *in vitro*. In addition, knowledge gaps and inconsistencies can be detected during the incorporation of new experimental results into the model, and additional questions are often raised following execution of the system model. A restriction of the approach is that due to lack of knowledge, the model might yield false positives, i.e., indicate that an erroneous mechanistic conjecture is correct. Hence, model-validated conjectures still need to be confirmed via wet-lab experiments. On the positive side, though, many such experiments can be avoided or refined if the model proves them wrong in the first place.

The model that we have developed is qualitative in nature; it relates to the temporal order and mechanisms underlying the biological system. It does not represent compartmental concentrations of reactants or kinetic coefficients that are required to formulate quantitative models. In order to construct and validate our qualitative mechanistic model, we have used simplified dynamics that treats each probabilistic experimental outcome as a Boolean outcome. The qualitative conceptual model may be the key for understanding the system and should serve as a prerequisite for designing quantitative modeling. During research and a requirement for publication, the researcher is required to suggest a qualitative abstract or schematic working model that is based on the quantitative probabilistic results obtained from experiments. However, the abstract models presented in publications may lead to incorrect conclusions because of lack of model formality or accidental misinterpretation of the biological outcome, knowledge gaps or wrong assumptions made in order to close knowledge gaps. The use of our model supports biologists as it enables construction of a coherent verifiable working model.

A major limitation of the proposed method is its scalability: It is not feasible to have human experts model “manually” knowledge embedded in thousands of scientific papers. Scientific knowledge is currently represented primarily as natural language text in Web-accessible scientific articles and reports that are interpretable exclusively by highly specifically trained humans. Preparing a consistent OPM model from 43 papers was a huge undertaking in itself. Therefore, we propose combining state-of-the-art natural language processing (NLP) and OPM, the emerging ISO 19450 standard, to address the system-wide problem of reading and integrating natural language text into OPM model-based, formally-organized knowledge that serves as a means for reasoning and hypothesis generation and testing. Based on experience gained in this kind of research we shall use the current model as a ‘ground truth’ model which will serve as the kernel to which knowledge in the form of model facts shall be incorporated. Intelligent NLP-aware crawlers shall continuously scavenger the Web for any newly published information related to knowledge already represented in the model and attempt to augment the model with it. Each model fact extracted from NLP deep semantic analysis shall undergo formal verification using model-checking techniques and, if needed, expert confirmation, prior to being incorporated into the model. This way we ensure the validity of the automatically evolving OPM model. Moreover, following this ‘*in-silico*’ testing we shall carry out actual wet-lab in vitro and in vivo experiments to confirm or refute conjectures and continuously evolve the ever-growing knowledge model.

Another limitation is that the conjecturing process aimed at filling knowledge gaps identified in the model requires experts to engage with the model and consult current literature. Indeed, it is hard to imagine that this stage of our process can be automated with current NLP technology. However, the mere identification of knowledge gaps is a major breakthrough in its own right, since currently it is done ad hoc, and not systematic as we propose.

Finally, the most valuable feature of our modeling system is its capacity to test the validity of biological hypotheses and pursue *in silico* experiments and obtain results that can later be examined experimentally. Indeed, investigators do such mental experiments routinely. However, our mental capacity is more limited than that of the computer, especially in handling complex systems. Moreover, unlike our mind, the computer is unbiased and is capable of proposing unexpected possibilities. Here we advocate the use of modeling systems, such as ours, to better analyze the enormous data that have been collected in biology.

## Materials and Methods

### 4.1 OPM

The mRNA decay model was constructed and executed with OPCAT (Object-Process Case Tool), which visually highlights each currently executing process, existing object, and current state. We used one instance defined for each object and one instance for each process. This enables qualitative analysis of the behavior of the biological system under study and its underlying mechanisms. During model execution, following a defined temporal order, the executor evaluates the precondition expression for each process. We used precondition links between objects/states and processes. If the precondition expression is evaluated to be true the related process is executed and if false the related process is skipped. Additional details on OPM and OPCAT for modeling molecular biology can be found at [3].

#### Objects

An object in the model represents a biological component (e.g., molecule, complex), a non-covalent association, or some attribute (e.g., a molecule location). An object can have different states, and at each time point it is in one of its states. Objects in the system that represent molecules, complexes or associations (i.e., **Links** or **Links Sets**) were defined as (having the state of) **existent** or **non-existent** (a shortcut used in the model is non-e). Molecular components such as binding domains can have other states such as **phosphorylated** or **dephosphorylated**.

The state of objects is changed during system execution. For example, when the mRNA is degraded, its state is changed from **existent** to **non-existent**. Another example is when two molecules dissociate; their ‘associating object’ is changed from **existent** to **non-existent**. Gene deletion is represented by initiating the object to be in its **non-existent** state. An example of an attribute is the location attribute object, such as **Xrn1 Location**, includes the states **nucleus** and **cytoplasm** and is initiated to be in the initial state **cytoplasm**.

#### Processes

A process represents a molecular function or a biological process assembled from a set of other biological processes or molecular functions. In our models, molecular functions are the basic, lowest level system processes, so they cannot be further in-zoomed. An example for a basic binding molecular function is **Ccr4-Not and Dhh1 Binding**. An example of a higher level biological process is **Decapping and Degradation** (see Figure 2).

#### Converting Quantitative Results to Qualitative Observations

*In vivo* and *in vitro* experimental results are in general quantitative in nature. Therefore, in order to evaluate our qualitative mechanistic model, we had to map the quantitative experimental results into qualitative observations. To this end, quantitative experimental outcomes are translated into corresponding qualitative Boolean values. For example, given that G1 is a gene and P1 is a biological process, then if the effect of deleting G1 on P1 activation was strong (but not necessarily total), we claim that G1 deletion → P1 activation = FALSE. Thus, a researcher may conclude that a strong, yet incomplete effect of deleting a given gene on the studied process indicates that this gene is responsible for the activation of this process, disregarding the fact that the effect is incomplete. Since our model is based on “all or nothing”, we model strong effects as being total even though they are often partial.

An example of a qualitative *in silico* experimental outcome that we used is as follows: deletion of **CCR4** and **Pan2** → impairment of **Deadenylation**. Such experiments are performed by changing the state of the object(s) to “**non-e**” (non-existent) and then checking the execution outcome. Thus, when both **CCR4** and **Pan2** are switched to “**non-e**”, the **Deadenylation** process should not be executed. Other observations, which were not related to process execution, referred to molecular localization, binding, or process temporal order. For example, the molecular localization observation “*Pab1 is imported into the nucleus*” is evaluated by checking that the object **Pab1 Location** indeed changes its state during execution from **plasma** into **nucleus**. An example of a binding observation is: *Rpb4/7 Binds Pat1*”, and an example of an observation related to process temporal order is “***Decaysome Import*** *occurs after* ***Degradation***”.

The qualitative interpretation of each experimental outcome was made based on the pertinent research papers suggesting the mechanistic “fact”. The qualitative interpretation of certain observations was undefined in the research paper. One example is the observation that the deadenylation rate in ccr4Δ cells is slower or “partially defective” [14, 15]. In this research paper, the probabilistic observation was not mapped into a Boolean value, and a working model was not established. Since deadenylation does occur in ccr4Δ cells, we modeled this observation to indicate that **Ccr4** deletion → **Deadenylation** occurs. As noted, we also modeled that deleting both **CCR4** and **Pan2** results in the impairment of **Deadenylation**.

### 4.2 Nuclear import assay

Cells from strains **yGH301** (*MATa, ade2, his3, leu2, trp1, ura3, mex67::HIS3 [pUN100 (CEN LEU2) mex67-5], xpo1::TRP1, xpo1-1::HIS3 [pMC476 (ADE2 CEN) PAB1-GFP]*) or **yGH444** (*MATa, ade2, his3, leu2, trp1, ura3, mex67::HIS3 [pUN100 (CEN LEU2) mex67-5], xpo1::TRP1, xpo1-1::HIS3 xrn1::ura3 [pMC570 (ADE2 CEN) xrn1^D208A^-GFP]*) were transformed with the centromeric plasmids pMC353 (*PAT1-RFP*); pMC270 (*LSM1-RFP*); pMC438 (*EDC3-RFP*) or pMC439 (*DCP1-RFP*). Cells were allowed to proliferate in synthetic complete media at 24°C, and subsequently diluted and allowed to proliferate (for at least 7 generations) to 3–5×10^6^ cells/ml. Cells were then incubated, with shaking, for 1 h at 37°C, at which point samples were taken for microscopy. For further details on this assay, plasmid construction, fluorescence microscopy and statistical analysis see [10].

## Supporting Information

File S1 **Supporting files. Table S1, Observations that our mRNA decay model is based on with their** ***in-silico*** **evaluations. Table S2, mRNA decay model assumptions and conjectures with explanations; these conjectures were required to create an executable model. Figure S1, Snapshot of model execution in** ***xrn1^D208A^*** **mutantion. Figure S2, Two hypothetical complexes, which our modeling system proposes (see** **Table 2** **) that comply with our experimental results.**
(DOC)

File S2 **Supporting videos. Video S1, A recording of mRNA decay model execution. Video S2, A recording of mRNA decay model execution for** ***PAT1*** **deletion.**
(ZIP)
