# Correction: Conceptual Modeling of mRNA Decay Provokes New Hypotheses

**DOI:** 10.1371/journal.pone.0114961

**Published:** 2014-12-02

**Authors:** The PLOS ONE Staff

There are errors in Figure 4. The authors have provided a corrected version here.

**Figure 4:**
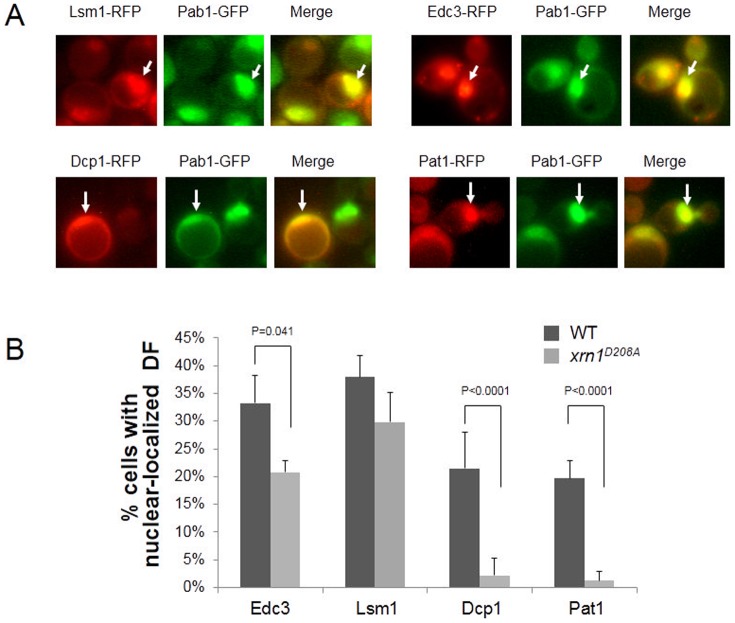
Experimental validation of model’s predictions shows that import of some decay factors is independent of Xrn1p exonuclease activity. XRN1 (WT) PAB1-GFP xpo1-1, mex67-5 cells, or xpo1-1, mex67-5, Δxrn1 cells expressing xrn1D208A-GFP and RFP fusion of the indicated DFs were proliferated at 24°C and then shifted to 37°C for 1 h; images were taken as previously described [10]. (A) Representative images of WT cells expressing the indicated proteins after 1h incubation at 37°C. Pab1-GFP, whose export is dependent on Xpo1p and Mex67p, serves as a nuclear marker, as described in [10]. Arrows point at examples of nuclei carrying both fluorescent proteins. All factors were cytoplasmic at 24°C ([10] and not shown) (B) Percentage of cells with nuclear localization of the indicated DF was determined, as described previously [10]. Mean values ± SD are shown. P-values of any pairwise difference that was <0.05 is indicated.
