# Supplementary material for: Conceptual Modeling of mRNA Decay Provokes New Hypotheses
Source: PLoS One. 2014 Sep 25;9(9):e107085. doi: 10.1371/journal.pone.0107085 (PMC4177816; doi:10.1371/journal.pone.0107085)
Supplement: File S1 — Supporting files. Table S1, Observations that our mRNA decay model is based on with their in-silico evaluations. Table S2, mRNA decay model assumptions and conjectures with explanations; these conjectures were required to create an executable model. Figure S1, Snapshot of model execution in xrn1D208A mutantion. Figure S2, Two hypothetical complexes, which our modeling system proposes (see Table 2 ) that comply with our experimental results. (DOC) [file pone.0107085.s001.doc]

Supporting Information for:

# Conceptual Modeling of mRNA Decay Provokes New Hypotheses

# Judith Somekh^ab*^, Gal Haimovich^bc^, Adi Guterman^bd^, Dov Dori^ae^ and Mordechai Choder^b^,

^a^Faculty of Industrial Engineering and Management, Technion - Israel Institute of Technology, Haifa 32000, Israel

^b^Faculty of Medicine, Technion - Israel Institute of Technology, Haifa 31096, Israel

^c^Current address: Albert Einstein College of Medicine, Yeshiva University, Bronx, New York 10461, USA

^d^Current address: Faculty of Biomedical Engineering, Technion - Israel Institute of Technology, Haifa 31096, Israel

^e^Engineering Systems Division, Massachusetts Institute of Technology, Cambridge, MA, USA

* Corresponding author

e-mail: ysomekh@gmail.com

This file includes:

Supporting Table S1-2

Supporting Figures S1-2

References

Table S1. Observations that our mRNA decay model is based on with their *in-silico* evaluations.

| **No.** | **Related**  **Process** | **Qualitative observation** | **Ref** | **In-silico Experiments/Evaluation of the observation on model execution** | **Underlying Wet Lab Experiment Type/Actual Experiment/Comments** |
| --- | --- | --- | --- | --- | --- |
|  | Rpb4/7 to Pat1 Binding | Rbp4/7 binds Pat1 | [1, 2] | **Rpb4/7-to-Pat1** Link Set object is initiated to **existent** | Physical Interaction |
|  | Rpb4/7 to RNA Binding | Rbp4 and Rpb7 each bind the mRNA 3' UTR | [In this paper] | **Rpb4/7-to-3`Area** Link Set object is initiated to **existent** | Physical Interaction |
|  | Pat1 Initiation | Pat1 binds the mRNA 3' UTR | [3] | **Pat1-to-3`Area** Link Set object is initiated to **existent** | Physical Interaction |
|  | Pat1 Recruitment | Pat1 binds RNA before deadenylation | [4, 5] | **Pat1-to-3`Area** object is initiated to **existent** before **Deadenylation** process is executed | [4] Physical Interaction: Pat1 co-IP with eIF4E, eIF4G and Pab1 (in RNase dependent manner) – meaning Pat1 binds mRNA before decapping and before deadenylation.  Lsm1 does not co-IP with any of these, indicating it binds the mRNA after deadenylation. |
|  | Translation: Pab1, eIF4G | Pab1 binds eIF4G | [6] | **Pab1-to-eIF4G** Link Set object is initiated to **existent** | Physical Interaction |
|  | Translation: eIF4F | eIF4E Binds eIF4G in the eIF4F complex | [6] | **eIF4G-to-eIF4E** Link Set object is initiated to **existent** | Physical Interaction |
|  | eIF4F to RNA Binding | eIF4F (cap binding complex) binds cap-RNA | [6] | **eIF4F-to-Cap** Link is initiated to **existent** | Physical Interaction |
|  | eIF4G to Dcp1 Binding | eIF4G binds Dcp1 | [21, 22] | **eIF4G Binds**  **Dcp1/2** process is executed changing the state of **eIF4F to Dcp1/2** Link Set into **existent** | Physical Interaction |
|  | Pab1 Import | Pab1 is imported into the nucleus | [7] | **Pab1 Import** process is executed, **Pab1 Location** object state is changed from **cytoplasm** into **nucleus** | Localization |
|  | Deadenylation:  Ccr4-Not | Ccr4-Not complex  And Pan2/3 degrade the poly A tail | [8, 9, 10] | **Ccr4** and **Pan2** deletion **🡪 Deadenylation** did not executed  **Ccr4** deletion **🡪 Deadenylation** executed  **Pan2** deletion **🡪 Deadenylation** executed | [9] Experiment: stop transcription and follow deadenylation by Northern analysis.  Ccr4 and Pan2 deletion 🡪deadenylation did not occur  Ccr4 deletion 🡪 deadenylation occurs (in cells – partial)  Pan2 deletion 🡪 deadenylation occurs (in cells – partial) |
|  | Deadenylation: Dhh1, Ccr4-Not | Dhh1 binds Ccr4-Not complex | [13] | **Dhh1 to Ccr4-Not**  **Binding** process is executed changing **Ccr4 Not-Dhh1 Link Set** into **existent** | Physical Interaction |
|  | Deadenylation, Decapping: Dhh1, Pat1 | Dhh1 Binds Pat1 | [14] | **Dhh1 and Pat1**  **Binding** process changes **Dhh1-to-Pat1 Link Set** object to existent | Physical Interaction (Co-IP experiments) |
|  | Lsm1-7 Recruitment to mRNA | LSM1-7 binds mRNA after deadenylation | [4] | The **LSM1-7 Complex Recruitment By Pat1 process** is executed after the **Deadenylation** process | [4] Physical Interaction: (1) Pat1 co-IP and Lsm1 does not co-IP with any of eIF4E, eIF4G and Pab1 (in RNase dependent manner), and (2) the Lsm proteins coimmunoprecipitate deadenylated mRNA. These two indicatesthat Lsm1-7 binds the mRNA after deadenylation. |
|  | Deadenylation, Decapping: Pat1 to Lsm1 Binding | Pat1 binds LSM1-7 | [15, 16, 17, 18] | **The LSM1-7 to Pat1 Link Set** is changed to **existent** | Physical Interaction |
|  | Deadenylation, Decapping: Pat1 to Lsm1 Binding | Lsm1-7 binds the C-terminal domain and middle regions of Pat1 | [14, 18] | **LSM1-7 Complex**  **Recruitment By Pat1** process is executed | Physical Interaction, Domains |
|  | Lsm1 Recruitment | Lsm1–7 remains associated with the mRNA 3`UTR after decapping and until the mRNA is fully degraded. | [4] | **Lsm1_7** and **3`RNA**  **Binding** process is executed after **Decapping** process.  **The LSM1-7 to 3`RNA Link Set** is changed to **non-existent** only after mRNA degradation. | Physical Interaction |
|  | Dcp2 Recruitment | Pat1-Lsm1-7 Complex recruits Dcp2 to mRNA | [17, 18, 4,  14] | **Pat1** OR **Lsm1-7** deletion **🡪 Pat1 to Dcp1 and Dcp2**  **Binding** and **Decapping** processes are not executed | [4] In vivo experiment: deletion of Pat1 reduces binding of Dcp1/2 to mRNA. (RNA –IP) Deletion of dcp1/2 or xrn1 does not affect recruitment of Pat1 or Lsm1 to mRNA (RNA-IP experiment).  Pat1 deletion 🡪No (less) recruitment of Dcp2 to the mRNA [4]  Lsm1 deletion 🡪 No (less) recruitment of Dcp2 to the mRNA [4]]  This is also suggested in the recent working model of Parker [37].  As Cited: “the decapping enzyme must be recruited to the mRNA…a working model is that the binding of [Pat1](http://www.yeastgenome.org/cgi-bin/locus.fpl?dbid=S000000673) to the mRNA with the [Lsm1](http://www.yeastgenome.org/cgi-bin/locus.fpl?dbid=S000003660)–7 complex allows for the formation of a binding site for [Dcp2](http://www.yeastgenome.org/cgi-bin/locus.fpl?dbid=S000005062) in [Pat1](http://www.yeastgenome.org/cgi-bin/locus.fpl?dbid=S000000673)’s C-terminal domain that is sufficient to activate catalysis” |
|  | Pat1 to Dcp1 /Dcp2 Binding Domains | Pat1 C-terminal domain (residues 422-796) binds Dcp1 and Pat1 C-terminal domain (residues 422-796) and Middle domain binds Dcp2 | [14, 18] | **Dcp1 to Pat1_D aa 422_697**  **Domains Binding** and **Dcp1 to Pat1_E aa 697_763**  **Domains Binding** and **Pat1 C-terminal Domain**  **to Dcp2 Binding** and **Pat1 Middle Domain to**  **Dcp2 Binding** and **Pat1 to Dcp2 Binding** processes are executed | Physical Interaction, Domains |
|  | Decapping | Dcp1/2 is activated by the Pat1-Lsm1-7 Complex, Dhh1 and Edc3 to bind and decapp the 5' cap structure of the mRNA | [4, 11, 12, 13] | **Pat1** deletion**🡪 Decapping** process is not **executed**  **Lsm1-7** deletion **🡪 Decapping** process is not executed  **Dhh1** deletion **🡪 Decapping** process is not executed  **Edc3** deletion**🡪 Decapping** is executed | Pat1 deletion🡪 No decapping  Lsm1-7 deletion 🡪 No decapping  Dhh1 deletion 🡪the cells accumulate capped mRNAs🡪 No decapping  (Dhh1 stimulates Dcp1/2 in vitro [13, 11]  Edc3 deletion 🡪 Decapping activated  (Edc3 does not inhibit decapping, unless dcp1 or dcp2 has mutations that make them less efficient [12].  Edc3 increases Dcp1/2 activity in vitro [12]). |
|  | Decapping: Dcp1/2 | Dcp1/2 is recruited to the 5` RNA region  prior to decapping | [20] | **Dcp1/2-to-5`Area** object changes states from **non-e** into **existent** prior to **Decapping.** | A complementary oligonucleotide was added to 5’, middle or 3’ end of capped-RNA substrate + Dcp1/2, in vitro. The decapping was inhibited only with the 5’ oligonucleotide. |
|  | Dcp1/2 structure | Dcp1 and Dcp2 form a heterodimer | [4, 21 23, 24] | **Dcp1_Dcp2 Complex** object is initiated to **existent** state, during system initiation | Physical Interaction |
|  | Decapping: Dcp1/2 | Dcp1/2 is the active heterodimer that decapp the mRNA | [23, 24] | **Dcp1** deletion **🡪 Decapping** process does not execute  **Dcp2** deletion **🡪 Decapping** process does not execute | Dcp1 deletion 🡪 decapping does not occur [24]  Dcp2 deletion 🡪 decapping does not occur [24]  Both in vitro and in vivo experiments |
|  | Edc3 Binding RNA | Pat1 binds Edc3 through the N-terminal LSM domain of Edc3p and C-terminal domain (residues 422-763) of Pat1p | [18] | **Pat1 to Edc3**  **Domain Binding** process is executed changing the state of **Edc3_N_Term to Pat1_C** object from **non-e** into **existent**. | Physical Interaction |
|  | Decapping | After decapping the cap (m7GDP) is removed from the RNA | [20, 24, 12] | **Dcp1/2** deletion **🡪 Decapping** process not executed, **Cap-to-RNA Link** object **= existent** | Dcp1/2 deletion 🡪 the cap was not removed from the mRNA [24] |
|  | Decapping: Edc3 | Edc3 participate in decapping | [12, 14] | **Edc3 deletion 🡪 Decapping process executed** | Edc3 deletion 🡪 Decapping occurs [12] |
|  | Decapping: Dhh1 | Dhh1 participate in decapping | [13, 11] | **Dhh1 deletion 🡪 Decapping process not executed** | Dhh1 deletion 🡪 No decapping |
|  | Decapping, Degradation | Decapping occurs before RNA degradation | [19] | **Decapping** process is executed before **5` to 3` Degradation** process | Xrn1 deletion 🡪 accumulation of un-capped mRNA |
|  | Decapping: Xrn1 | Xrn1 is not required for Decapping | [19, 1213] | **Xrn1 object** initiated to **non-existent 🡪 Decapping** occurs,  Xrn1 deletion 🡪 RNA is un-capped | Xrn1 deletion 🡪 (uncapped RNA accumulated) Decapping occurs |
|  | Decapping: Xrn1 | Xrn1 binds 5'-p-RNA after decapping | [26] | **Xrn1 to 5`RNA**  **Binding** process is executed after **Decapping** process is executed |  |
|  | Degradation: Xrn1 | Xrn1 active site degrades RNA | [26, 27] | **Xrn1 Active Site** deletion **🡪 Xrn1 Degrade RNA from 5` end** process not executed | Active site disruption 🡪 Xrn1 does not degrade RNA |
|  | Degradation: Xrn1 | 5'-p-RNA binding site is needed for binding of Xrn1 to RNA | [26] | **Xrn1 5` phosphate RNA** deletion **🡪 Xrn1 to 5`RNA Binding** process is not executed | 5'-p-RNA binding site deletion 🡪 Xrn1 does not bind RNA |
|  | Degradation: Xrn1 | Xrn1 active site and 5'-p-RNA binding sites are required for degradation of the RNA by Xrn1 | [29] | **Xrn1 5` phosphate RNA** deletion OR **Xrn1 Active Site** deletion**(🡪 Xrn1 Degrade RNA from 5` end** process not executed | 5'-p-RNA binding site mutation or active site mutation🡪 Xrn1 does not degrade the RNA |
|  | Xrn1 Recruitment | Pat1 C-terminal domain binds Xrn1 | [17, 14] |  | Physical Interaction, Domain |
|  | Degradation: Xrn1, Dcp1/2 | Xrn1 binds Dcp1/2 | [30, 31] | **Dcp1/2-to-Xrn1 Link Set is** change to **existent** by the **Dcp1/2 Binds Xrn1**  process | Physical Interaction |
|  | Degradation: Xrn1 | Xrn1 degrades RNA after binding to it | [26, 28, 19] | Deletion of **5` phosphate RNA Binding domain** of **Xrn1 🡪 5` to 3`**  **Degradation** process is not executed | Mutations of residues involved in binding the 5′-terminal nucleotide impair Xrn1 processivity |
|  | Degradation: Exosome | Exosome, alternatively to Xrn1, degrades RNA after deadenylation | [41, 42] | **Xrn1** and **Exosome** deletion **🡪 5` to 3`**  **Degradation** process is not executed**, 3` to 5`**  **Degradation** process is not executed**.**  **Xrn1** deletion **🡪 3` to 5`**  **Degradation** process is executed  **Exosome** deletion **🡪 5` to 3`**  **Degradation** process is executed | Xrn1 and Exosome deletion 🡪 No Degradation, and ***Cells are not viable.***  Xrn1 deletion 🡪 No 5`-to-3` degradation  Exosome deletion 🡪 No 3`-to-5` degradation |
|  | Decaysome Import | Decaysome Import occurs after Degradation | [29] | **Decaysome Import** process is executed after **Degradation** process | All DFs are imported into the nucleus. Import is inhibited in cells carrying an enzyme dead Xrn1p. This inhibition is alleviated when a 2nd mutation at Xrn1 binding pocket is created. |
|  | Xrn1 Import | Xrn1 imports into the nucleus | [29] | **Xrn1 Import** process executed, **Xrn1 Location** object state is changed from **cytoplasm** into **nucleus** | Localization |
|  | Decaysome Import | if Xrn1 binds RNA but doesn't degrade it, Dcp1/2 does not import into the nucleus | [29] | **Xrn1 Active Site** object initiated to **non-existent 🡪 Dcp1/2 Complex Import process** is not executed, **Xrn1 Import** is not executed | Xrn1 active site disruption 🡪 Dcp1/2 does not import, Xrn1 does not import |
|  | Decaysome Import | if Xrn1 does not bind RNA, both Xrn1 and Dcp1/2 are imported to the nucleus | [29] | **Xrn1 5` Phosphate RNA object** initiated to non-existent **🡪 Dcp1_Dcp2 Complex Import process** executed**, Xrn1 Import** process executed | Xrn1 5`RNA binding site deletion 🡪 Dcp1/2 imports, Xrn1 imports (partially) |
|  | Decaysome Import | If Xrn1 is deleted, Dcp2 imports into the nucleus | [29] | **Xrn1** object initiated to **non-existent 🡪 Dcp2 Complex Import** process executed | Xrn1 deletion 🡪 Dcp2 imports |
|  | Decaysome Import | CCr4-Not, Rpb4/7, Pat1, Dcp1/2, Lsm1, Xrn1, Dhh1, Edc3 import into the nucleus | [[39, 43] shows Rpb4 import, [29] shows CCr4-Not, Pat1, Dcp1/2, Lsm1, Xrn1, Dhh1 and Edc3 import] | The import process is executed for each factor, **Location** object state is changed from **cytoplasm** into **nucleus** for each factor | Localization |
|  | Decaysome Import | Lsm1 and Edc3 import into the nucleus independently of Xrn1  Dcp1/2 and Pat1 is dependent on Xrn1 activity | [This work, for Dcp2 see [29]] | **Xrn1 active site** deletion **🡪 Lsm1** and **Edc3** import into the nucleus, **Dcp1/2** and **Pat1** does not import | Xrn1 active site D^208A^ mutant 🡪 Lsm1 and Edc3 import into the nucleus, Dcp1/2 and Pat1 does not import |

Table S2. mRNA decay model assumptions and conjectures with explanations; these conjectures were required to create an executable model.

| **No.** | **Related**  **Process** | **Conjecture incorporated into the model** | **Related Experimental observation [ref.]** | **Conjecture Explanation (what is the basis for the conjecture)** | **In-silico Experiment** |
| --- | --- | --- | --- | --- | --- |
|  | Lsm1-7 recruitment | Pat1p recruits Lsm1-7 to mRNA | [15, 16, 17, 18] | We agree with the suggestion of Nissan & Parker [14] and conjecture that Pat1p recruits Lsm1-7 to the RNA since – (1)The Lsm1–7 complex is associated with Pat1p [14, 15, 16, 17, 18], (2) Pat1p is required for the recruitment of Lsm1 to P bodies [40] and (3) the interaction between Pat1 and Lsm1-7 is dependent on the C-terminal domain of Pat1p both in P-bodies [18] and in the cytoplasm [14]. Thus the mechanism found in P-bodies may be similar to the mechanism in the cytoplasm. | **Pat1** deletion 🡪 **Lsm1-7** is not recruited to the RNA |
|  | Pre-decapping | Pat1p recruits LSM1-7 after deadenylation. | Pat1p recruits LSM1-7 [4, 15] | Lsm1p does not co-IP with eIF4E, eIF4G or Pab1p (in RNase dependent manner) [4], indicating it binds the mRNA after deadenylation. | **Lsm1-7 Complex Recruitment by Pat1** process is executed after **Deadenylation** process |
|  | Pab1p import | Pab1p is imported into the nucleus after deadenylation. Its prior association with the Poly(A) tail is prerequisite for its import. | Pab1p dissociates from eIF4G after deadenylation [4]  Pab1 imports into the nucleus [7] | Pab1p imports into the nucleus after deadenylation that releases Pab1p from the RNA and after Pab1p dissociates from eIF4G (as both poly(A) tail and eIF4G would retain Pab1p in the cytoplasm). Pab1 is exported in complex with the Poly(A) tail. Hence we hypothesize that prior association with the Poly(A) tail is prerequisite for its import. | **Pab1 Import** process is executed after **Deadenylation** process. Pre-condition for **Pab1 Import** is that the object **Pab1-to-Poly A tail** is in its **non-e** (non-existent) state |
|  | Deadenylation | Dhh1p and Ccr4Not bind when the model is initiated. | Dhh1p binds Ccr4Not [13, 32]  These two partners form complex during transcription elongation [33]  These two partners were found to import after mRNA decay [29] | Since there is no reason to assume otherwise, we assume that Dhh1 and Ccr4Not are bound to each other throughout the decay process. | **Dhh1 to Ccr4Not Binding** process is executed |
|  | Deadenylation, Decapping | Dhh1p and Pat1p form a complex throughout the decay process. | Dhh1p binds Pat1p [14]  Dhh1 participates in decapping [13]  Pat1 binds the RNA before deadenylation [4, 5] | Since there is no reason to assume otherwise we assume that Dhh1p and Pat1p bind each other throughout the decay process. | **Dhh1 and Pat1 Binding** process is executed during **System Initiation** process |
|  | Pre-decapping | Pat1p binds Xrn1p before deadenylation. | Pat1p binds Xrn1p [17, 14] | Since Xrn1p was described to be associated with polysomes [34, 35] we assume that Pat1p binds Xrn1p before deadenylation. | **Pat1 Binds Xrn1** process is executed before **Deadenylation** process |
|  | Pre-decapping | eIF4F must dissociate from the cap-RNA before Dcp2p binds cap-RNA. | eIF4F (cap binding complex) binds cap-RNA [6]  eIF4F dissociate cap-RNA [36]  Dcp2 binds cap-RNA [24] | Both factors bind the same cap. Crystal structure shows that eIF4E, a component of eIF4F, engulfs the entire cap [36]. Therefore, the two factors cannot bind the cap simultaneously. | **eIF4F Release** process is executed before the **Dcp2 to Cap**  **Binding** process is executed |
|  | Pre-decapping | Pat1p binds Edc3p, during pre-decapping assembly (before decapping and after Lsm1-7 recruitment). | Pat1p binds Edc3p [14, 18] | Edc3p enhances decapping [12] and should be recruited before decapping.  Lsm1p and Edc3p bind different Pat1 domains [18]. The timing dependencies between the recruitment of Lsm1-7 and Edc3p are unknown. We arbitrarily assume that Lsm1-7 is recruited first. Importantly, there is no effect on the executable results if we assume otherwise. | **Pat1 to Edc3**  **Domains Binding** process is executed before **Decapping** process and after **LSM1-7 Complex**  **Recruitment By Pat1** process |
|  | Decapping | Dcp1p and Dcp2p form a heterodimer throughout the decay process. | Dcp1p and Dcp2p form a heterodimer [4, 21, 23, 24] | Since there is no reason to assume otherwise, we assume that Dcp1/2 remains a heterodimer throughout the decay process. | **Dcp1/2** complex is **existent** throughout the decay process |
|  | Decapping | Dcp1/2 dissociates from mRNA or the decaysome after decapping.  *** Our framework disagreed with this common conjecture.** | Dcp1/2 dissociate RNA after decapping [21, 23, 24, 37, 38] | This common assumption [37, 38] that Dcp1/2 dissociated after decapping was refuted by our model (see next row for refined conjecture). |  |
|  | RNA degradation and decaysome import | Dcp1/2 stays bound to the decaysome after decapping. | Dcp1/2 binds to a long stretch of nucleotides at the 5’ region of the RNA prior to decapping [‎20]. Following degradation of this region, we propose that it is bound to the decaysome. | We assume that Dcp1/2 remains associated with the decaysome until complete mRNA degradation, which stimulates its import together with Xrn1p. | **During Pat1 Recruits Dcp1/2**  **To mRNA** process execution, the **Dcp1/2 to 5`RNA**  **Recruiting** process is executed |
|  | Decaysome import | CCr4-Not, Pat1p, Lsm1p, Edc3p, Xrn1p, Dcp1/2 and Dhh1p dissociate and import into the nucleus independently.  ***Our framework disagreed with this conjecture.** |  | Due to lack of knowledge we initially assumed that each decay factor import is carried out concurrently and independently. As we explain in the paper, this conjecture was refined (see next row, row 17 and explanation in the paper). | During **Decaysome**  **Import** process each factor imports independently |
|  | RNA degradation and decaysome import | Dcp1/2 remains bound to Xrn1p after its recruitment (before decapping) and during the entire RNA degradation process and during import. | Xrn1p and Dcp1/2 bind each other [30, 31]  If the enzyme dead Xrn1 binds RNA, both Xrn1p and Dcp2p do not import into the nucleus [29]  If the capacity of the enzyme dead Xrn1 to bind the RNA 5`end is compromised, both Xrn1 and Dcp2 import into the nucleus [29]  If the capacity of Xrn1 to bind the RNA 5`end is compromised, both Xrn1 and Dcp2 import into the nucleus [29]  If Xrn1 is deleted then Dcp2 imports into the nucleus [29] | These observations confirm that import of Xrn1p and Dcp1/2 is dependent on their interaction.  Since there is no reason to assume otherwise, we assume that they physically interact, after Dcp1/2 recruitment before decapping by Pat1p, during the degradation process, remain bound after degradation completed and are imported together thereafter. | **Dcp1_2 Binds**  **Xrn1** process is executed before **Decapping** process |
|  | RNA degradation and decaysome import | Xrn1p remains bound to Pat1p after its recruitment to the 5`end of the RNA and during import. | Pat1p binds Xrn1p [17, 14]  Xrn1 binds 5'-p-RNA after decapping [26] | Since there is no reason to assume otherwise, and because Pat1 import is dependent on Xrn1 import (Fig. 4), we assume that after its recruitment, Xrn1p remains permanently bound to Pat1p. | **Xrn1-to-Pat1** Link set object is **existent** through model execution |
|  | RNA degradation and decaysome import | Xrn1p degrades the mRNA and releases all factors from their binding to the mRNA. | [4, this work] | According to our experiment (see Fig. 3) the DFs seem to stay attached to the mRNA after deadenylation and decapping, and after 5’ to 3’ exonucleolytic degradation has begun. This result supports our conjecture.  Indeed, [4] supports this conjecture for Lsm1-7. It was found that the Lsm1p–7p complex remains associated with the 3′ UTR of the mRNP after decapping and during the exonucleolysis by Xrn1p until the mRNA is fully degraded. | **Xrn1 Releases**  **Decaysome** process is executed after **Xrn1 Degrades RNA** process |
|  | Decaysome import | Each of CCr4Not, Pat1p, Lsm1p, Edc3p, Xrn1-Dcp1/2 Complex and Dhh1p bind to an unknown distinct import factor. | Rpb4p has an active NLS [39] | Due to lack of knowledge about the import mechanism we assume that each decay factor has a distinct import factor and has the capability to import independently (if it is released from the RNA). | The relevant processes representing the binding to the import factor are executed |
|  | Decaysome import | RNA binding is prerequisite for import. | import executed only after mRNA degradation [29] and decay factors releasing from RNA [29 and in this paper] | Import of Xrn1 occurs only following its recruitment to the RNA [29]. Based on this precedence, we hypothesize that RNA binding is prerequisite for import other DFs. | Specified through the model |
|  | RNA degradation and decaysome import | Xrn1p imports in the context of at least Dcp1/2 and Pat1p | If the capacity of Xrn1p to bind the RNA 5`end is compromised, Xrn1p and Dcp2p import into the nucleus [29]  If both the capacity of Xrn1p to bind the RNA 5`end and its enzymatic activity are disrupted, Xrn1p and Dcp2p import into the nucleus [29]  If Xrn1p is deleted, Dcp2p imports into the nucleus [29] | According to our previous findings (summarized in Table 1), import of Dcp2p is dependent on Xrn1p. This implies that they are imported together as a complex. Here we show that Pat1p import is dependent on Xrn1p. The rest of the decay factors were found to import [29] but there is lack of knowledge regarding their associations with other factors during their import. Thus, except from the Xrn1-Dcp1/2-Pat1, we tentatively assumed that all the decay factors are import independently. | Xrn1p-Dcp1/2-Pat1p are modeled to import as one complex |
|  | RNA degradation and decaysome import | Xrn1p and its associated molecules (specifically Dcp1/2 and Pat1p) are not imported as long as Xrn1p active site contains RNA. | [29] | If the capacity of Xrn1p to bind the RNA 5`end is compromised by mutations, Xrn1p and Dcp2p import into the nucleus [29]  If both the capacity of Xrn1p to bind the RNA 5`end and its enzymatic activity are disrupted, Xrn1p and Dcp2p import into the nucleus [29]  If Xrn1p is deleted then Dcp2p imports into the nucleus [29]  If the Xrn1p active site is mutated, Xrn1p, Dcp2p [29] and Pat1p [shown in this work] do not import into the nucleus. | Specified through the model |


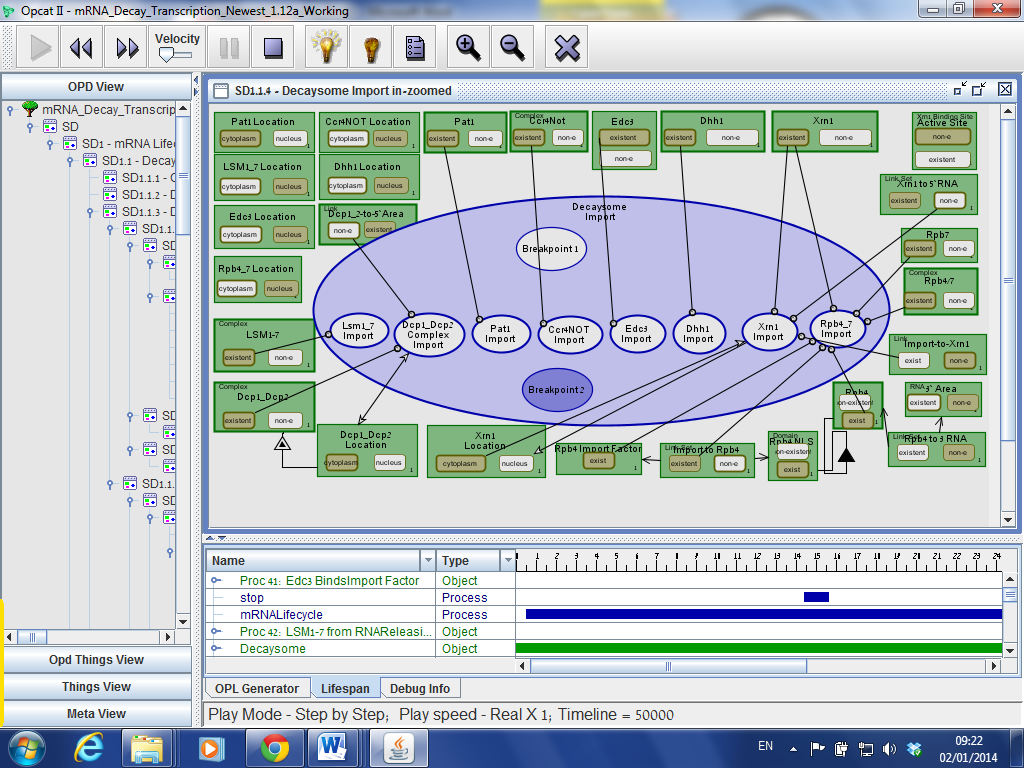


Figure S 1. Snapshot of model execution in *xrn1^D208A^* mutantion. It can be seen that when Xrn1 active site is deleted (highlighted in red) then Pat1p, Xrn1p and Dcp1/2 are not imported into the nucleus (their state, highlighted in blue, is “cytoplasm” and not “nucleus” after the Decaysome Import process occurred) and Lsm1-7 and Edc3 are imported into the nucleus.


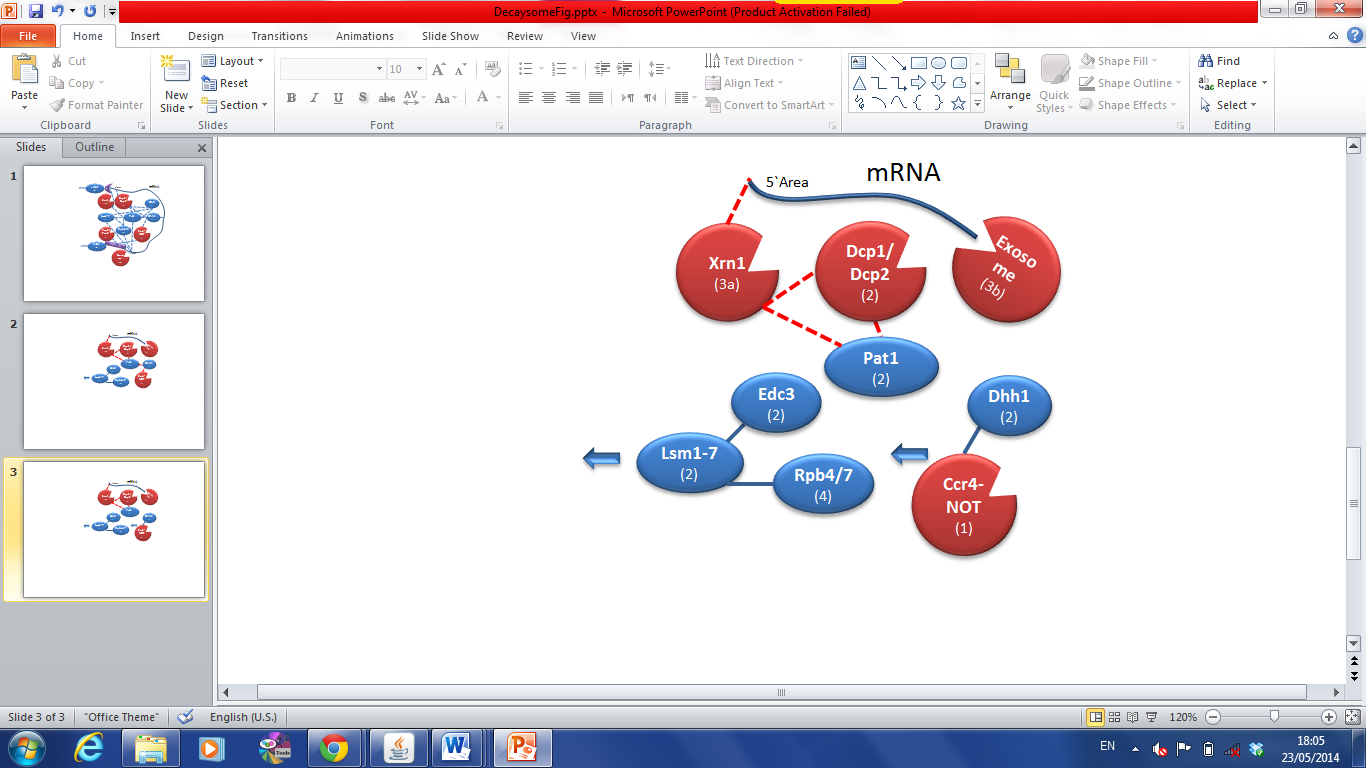

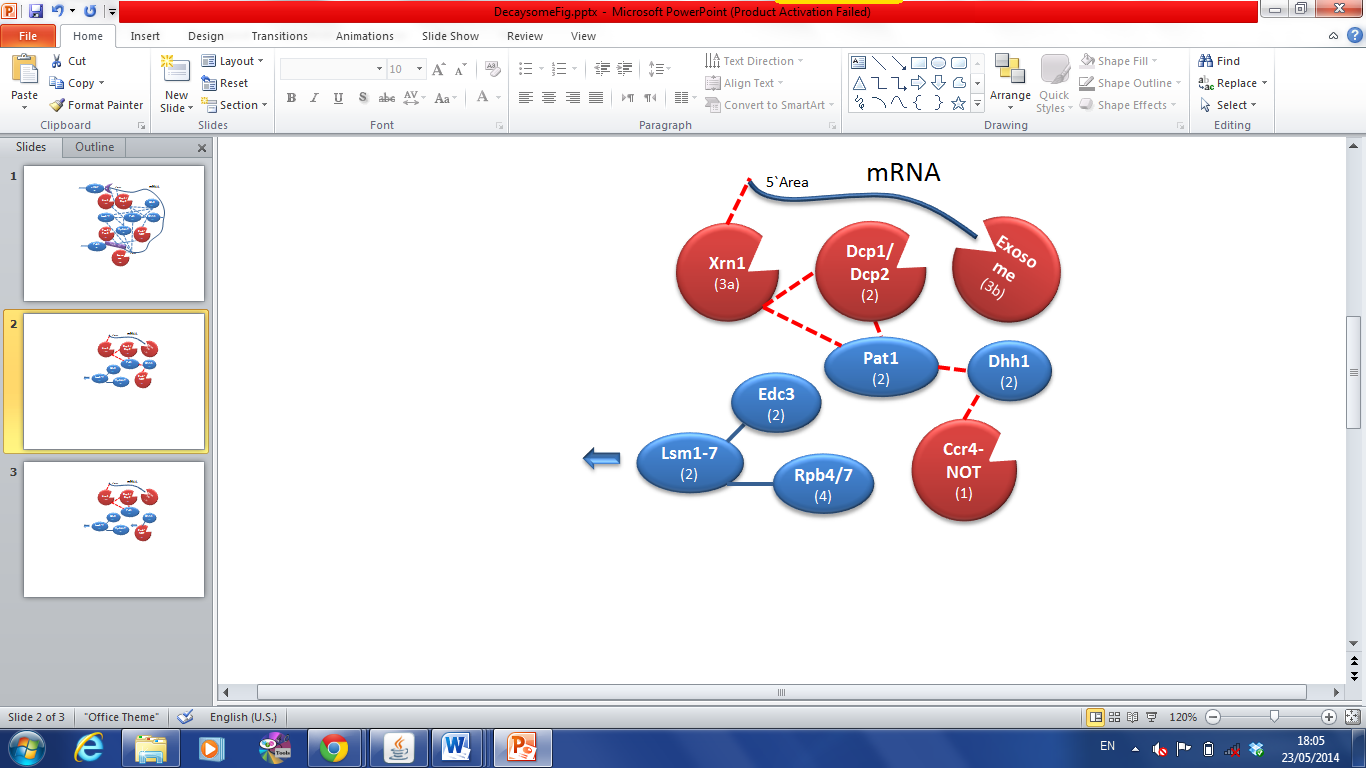


1. (B)

Figure S2. Two hypothetical complexes, which our modeling system proposes (see Table 2) that comply with our experimental results. There are 4096 (2^12^) possibilities for the transient interaction network of the decay factors. We show here two possibilities that were verified by the model to match the experimental findings, out of 11 that we chose to execute (see Table 2). (A) In this option there exists three distinct complexes; Xrn1p-Dcp1/2-Pat1p, Dhh1-Ccr4Not and Edc3p-Lsm1-7-Rpb4/7 (matches column 4 in Table 2). (B) In this option two distinct complexes exist: Xrn1p-Dcp1/2-Pat1p-Dhh1-Ccr4Not and Edc3p-Lsm1-7-Rpb4/7 (matches column 6 in Table 2).

# References

1. Lotan, R., Bar-On, V.G., Harel-Sharvit, L., Duek, L., Melamed, D., and Choder, M. (2005). The RNA polymerase II subunit Rpb4p mediates decay of a specific class of mRNAs. Genes Dev 19, 3004-3016.
2. Lotan R., Goler-Baron V., Duek L., Haimovich G., Choder M. (2007). The Rpb7p subunit of yeast RNA polymerase II plays roles in the two major cytoplasmic mRNA decay mechanisms. J Cell Biol 178(7): 1133-1143.
3. Chowdhury, A., Mukhopadhyay, J., Tharun, S. (2007). The decapping activator Lsm1p-7p–Pat1p complex has the intrinsic ability to distinguish between oligoadenylated and polyadenylated RNAs. Rna, 13(7), 998-1016.
4. Tharun, S., Parker, R. (2001). Targeting an mRNA for decapping: Displacement of translation factors and association of the Lsm1p–7p complex on deadenylated yeast mRNAs. Molecular Cell, 8(5), 1075-1083.
5. Bonnerot, C., Boeck, R., and Lapeyre, B. (2000). The two proteins Pat1p (Mrt1p) and Spb8p interact in vivo, are required for mRNA decay, and are functionally linked to Pab1p. Mol Cell Biol 20, 5939-5946.
6. Jackson, R. J., Hellen, C. U. T., Pestova, T. V. (2010). The mechanism of eukaryotic translation initiation and principles of its regulation. Nature Reviews Molecular Cell Biology, 11(2), 113-127.
7. Brune C, Fischer N, Podtelejnikov AV, Weis K. (2005). Yeast poly(A)-binding protein Pab1 shuttles between the nucleus and the cytoplasm and functions in mRNA export. RNA 11: 517-531.
8. Tucker, M., M. A. Valencia-Sanchez, et al. (2001). The transcription factor associated Ccr4 and Caf1 proteins are components of the major cytoplasmic mRNA deadenylase in Saccharomyces cerevisiae. Cell 104(3): 377-386.
9. Tucker, M., R. R. Staples, et al. (2002). "Ccr4p is the catalytic subunit of a Ccr4p/Pop2p/Notp mRNA deadenylase complex in Saccharomyces cerevisiae." Embo J 21(6): 1427-1436.
10. Lowell JE, Rudner DZ, Sachs AB (1992). 3'-UTR-dependent deadenylation by the yeast poly(A) nuclease. Genes Dev. Nov;6(11):2088-99.
11. Fischer, N., and Weis, K. (2002). The DEAD box protein Dhh1 stimulates the decapping enzyme Dcp1. Embo J 21, 2788-2797.
12. Kshirsagar, M., and Parker, R. (2004). Identification of Edc3p as an enhancer of mRNA decapping in Saccharomyces cerevisiae. Genetics 166, 729-739.
13. Coller, J.M., Tucker, M., Sheth, U., Valencia-Sanchez, M.A., and Parker, R. (2001). The DEAD box helicase, Dhh1p, functions in mRNA decapping and interacts with both the decapping and deadenylase complexes. Rna 7, 1717-1727.
14. Nissan, T., Rajyaguru, P., She, M., Song, H., and Parker, R. (2010). Decapping activators in Saccharomyces cerevisiae act by multiple mechanisms. Mol Cell 39, 773-783.
15. Tharun, S., He, W., Mayes, A.E., Lennertz, P., Beggs, J.D., and Parker, R. (2000). Yeast Sm-like proteins function in mRNA decapping and decay. Nature 404, 515-518.
16. Coller J. and Parker, R. (2004). Eukaryotic mRNA decapping. Annu. Rev. Biochem. 73: 861-890.
17. Bouveret, E., Rigaut, G., Shevchenko, A., Wilm, M., & Séraphin, B. (2000). A sm-like protein complex that participates in mRNA degradation. The EMBO Journal, 19(7), 1661-1671.
18. Pilkington, G. R., Parker, R. (2008). Pat1 contains distinct functional domains that promote P-body assembly and activation of decapping.  Molecular and Cellular Biology, 28(4), 1298-1312.
19. Hsu, C.L., and Stevens, A. (1993). Yeast cells lacking 5'-->3' exoribonuclease 1 contain mRNA species that are poly(A) deficient and partially lack the 5' cap structure. Mol Cell Biol 13, 4826-4835.
20. Steiger, M., Carr-Schmis, A., et. al. (2003). Analysis of recombinant yeast decapping enzyme. Rna, 9(2), 231-238.
21. Vilela, C., C. Velasco, et al. (2000). The eukaryotic mRNA decapping protein Dcp1 interacts physically and functionally with the eIF4F translation initiation complex. Embo J 19(16): 4372-4382.
22. Ramirez CV, Vilela C, Berthelot K, McCarthy JE. (2002) Modulation of eukaryotic mRNA stability via the cap-binding translation complex eIF4F. J Mol Biol 318: 951-962.
23. Deshmukh, M.V., Jones, B.N., Quang-Dang, D.U., Flinders, J., Floor, S.N., Kim, C., Jemielity, J., Kalek, M., Darzynkiewicz, E., and Gross, J.D. (2008). mRNA decapping is promoted by an RNA-binding channel in Dcp2. Mol Cell 29, 324-336.
24. Dunckley, T., and Parker, R. (1999). The DCP2 protein is required for mRNA decapping in Saccharomyces cerevisiae and contains a functional MutT motif. EMBO J 18, 5411-5422.
25. Liu, H., and Kiledjian, M. (2005). Scavenger decapping activity facilitates 5' to 3' mRNA decay. Mol Cell Biol 25, 9764-9772.
26. Jinek, M., Coyle, S.M., and Doudna, J.A. (2011). Coupled 5' nucleotide recognition and processivity in Xrn1-mediated mRNA decay. Mol Cell 41, 600-608.
27. Solinger JA, Pascolini D, Heyer W. (1999) Active-site mutations in the Xrn1p exoribonuclease of saccharomyces cerevisiae reveal a specific role in meiosis. Mol Cell Biol 19: 5930-5942.
28. Stevens, A. (2001). 5'-exoribonuclease 1: Xrn1. Methods Enzymol 342, 251-259.
29. Haimovich G, Medina DA, Causse SZ, Garber M, Millán-Zambrano G, Barkai O, et al. Gene expression is circular: Factors for mRNA degradation also foster mRNA synthesis. Cell. 2013;153(5):1000-11.
30. Braun, J. E., Truffault, V., Boland, A., Huntzinger, E., Chang, C., Haas, G., Izaurralde, E. (2012). A direct interaction between DCP1 and XRN1 couples mRNA decapping to 5′ exonucleolytic degradation. Nature Structural & Molecular Biology.
31. Fromont Racine, M., Mayes, A. E., Brunet-Simon, A., Rain, J. C., Colley, A., Dix, I., et al. (2000). Genome wide protein interaction screens reveal functional networks involving Sm-like proteins. Yeast, 17(2), 95-110.
32. Hata, H., Mitsui, H., Liu, H., Bai, Y., Denis, C. L., Shimizu, Y., et al. (1998). Dhh1p, a putative RNA helicase, associates with the general transcription factors Pop2p and Ccr4p from saccharomyces cerevisiae. Genetics,148(2), 571-580.
33. Kruk, J.A., Dutta, A., Fu, J., Gilmour, D.S., and Reese, J.C. (2011). The multifunctional Ccr4-Not complex directly promotes transcription elongation. Genes Dev 25, 581-593.
34. Mangus D.A. and Jacobson, A. (1999) Linking mRNA turnover and translation: assessing the polyribosomal association of mRNA decay factors and degradative intermediates. Methods, 17, 28–37.
35. Hu, W., Sweet, T. J., Chamnongpol, S., Baker, K. E., Coller, J. (2009). Co-translational mRNA decay in saccharomyces cerevisiae. Nature, 461(7261), 225-229.
36. Schwartz, D.C., and Parker, R. (2000). mRNA decapping in yeast requires dissociation of the cap binding protein, eukaryotic translation initiation factor 4E. Mol Cell Biol 20, 7933-7942.
37. Parker, R. (2012). RNA degradation in Saccharomyces cerevisae. Genetics 191(3): 671-702.
38. Garneau, N. L., Wilusz, J., Wilusz, C. J. (2007). The highways and byways of mRNA decay. Nature Reviews Molecular Cell Biology, 8(2), 113-126.
39. Harel-Sharvit L, Eldad N, Haimovich G, Barkai O, Duek L, Choder M. (2010). RNA polymerase II subunits link transcription and mRNA decay to translation. Cell. 143(4):552-63.
40. Teixeira D, Parker R. (2007) Analysis of P-body assembly in saccharomyces cerevisiae. Mol Biol Cell 18: 2274-2287.
41. Johnson AW, Kolodner RD. (1995) Synthetic lethality of sep1 (xrn1) ski2 and sep1 (xrn1) ski3 mutants of saccharomyces cerevisiae is independent of killer virus and suggests a general role for these genes in translation control. Mol Cell Biol 15: 2719-2727.
42. Anderson JSJ, Parker R. (1998) The 3′ to 5′ degradation of yeast mRNAs is a general mechanism for mRNA turnover that requires the SKI2 DEVH box protein and 3′ to 5′ exonucleases of the exosome complex. EMBO J 17: 1497-1506.
43. Selitrennik M, Duek L, Lotan R, Choder M. (2006) Nucleocytoplasmic shuttling of the Rpb4p and Rpb7p subunits of saccharomyces cerevisiae RNA polymerase II by two pathways. Eukaryotic cell 5: 2092-2103.
